# Assessment of innovative living and care arrangements for persons with dementia: a systematic review

**DOI:** 10.1186/s12877-023-04187-4

**Published:** 2023-08-01

**Authors:** C. Speckemeier, A. Niemann, M. Weitzel, C. Abels, K. Höfer, A. Walendzik, J. Wasem, S. Neusser

**Affiliations:** Institute for Healthcare Management and Research, University of Duisburg-Essen, Thea-Leymann-Str. 9, 45127 Essen, Germany

**Keywords:** Dementia, Long-term care, Living environment, Innovative, Quality of life, Review

## Abstract

**Background:**

Alternative forms of housing for persons with dementia have been developed in recent decades. These concepts offer small groups of residents familiar settings combined with efforts to provide normal daily life. The aim of this systematic review is to collate and analyze these more innovative forms of housing regarding residents’ quality of life, behavioral aspects, as well as functional, cognitive and emotional aspects.

**Methods:**

Searches were conducted in PubMed, EMBASE and PsycInfo in November 2020. Studies comparing traditional and more innovative living environments for persons with dementia were eligible. Concepts are described based on the results of additional searches. Risk of bias of included studies was assessed using checklists from the Joanna Briggs Institute.

**Results:**

A total of 21 studies corresponding to 11 different concepts were included, namely Green Houses (USA), Group Living (Sweden), Cantou (France), Group Homes (Japan), Small-scale Group Living (Austria), Special Care Facilities (Canada), Shared-housing Arrangements (Germany), Residential Groups (Germany), Residential Care Centers / Woodside Places (USA/Canada), Small-scale Living (Netherlands/ Belgium), and Green Care Farms (Netherlands). The concepts are broadly similar in terms of care concepts, but partly differ in group sizes, staff qualifications and responsibilities. Several studies indicate that innovative forms of housing may encourage social behavior, preserve activity performance and/or positively influence emotional status compared to more traditional settings, while other studies fail to demonstrate these effects. Some studies also show increased behavioral and psychological symptoms of dementia (BPSD) in residents who live in more innovative housing concepts. The effect on cognition remains indistinct.

**Discussion:**

The positive effects may be attributable to the inherent characteristics, including small group sizes, a stimulating design, and altered staff roles and responsibilities. Arguably, some of these characteristics might also be the reason for increased BPSD. Studies had variable methodological quality and results have to be considered with caution. Future research should examine these effects more closely and should investigate populations’ preferences with regards to housing in the event of dementia.

**Supplementary Information:**

The online version contains supplementary material available at 10.1186/s12877-023-04187-4.

## Background

Around 55 million people are affected by dementia worldwide [1]. Although age-specific incidence rates have declined over the past 25 years [2], the number of persons with dementia is projected to rise in the coming years due to a steadily increasing life expectancy [3, 4]. Dementia is known to have psychological, physical, social and economic impact on persons with dementia, their caregivers and the society [1]. In the early stage of dementia, a person usually functions independently. Medium dementia is typically the longest phase and can last for many years. Persons with severe dementia lose the ability to respond to their environment appropriately and need extensive care [5].

Dementia results in a heterogeneous range of behavioral and psychological symptoms (BPSD), which pose a source of distress on caregivers and the person with dementia [6]. Consequently, domestic care of persons with dementia is more difficult, time-consuming and stressful compared to care of persons without dementia [7]. BPSD are a major reason for earlier institutionalization of patients [6]. In a study determining the prevalence of BPSD in long-term care environments, 79% of persons with dementia had clinically significant BPSD. Interestingly, prevalence of clinically significant BPSD did not differ between residents with different severity of dementia [8]. It is thought that only 10% of nursing home residents’ symptoms are caused by dementia itself, while 90% result from the quality of care which persons with dementia receive [9]. According to Nazarko (2009), provision of a good quality of care requires person-centered care and a stimulating environment [9]. BPSD may therefore be a means to express a lack of social support or discomfort [10].

A large proportion of people with dementia are admitted to long-term care facilities during the course of their disease, which may be due to BPSD or exhaustion of the caregiver [11]. Traditionally, persons with dementia reside in institutions without an emphasis on the clinical picture of dementia; rather, the provision of care follows a medical-somatic approach [12]. Since the mid-1990s, institutional care of older adults has changed regarding physical environment, staff roles and processes [13]. Special Care Units (SCUs) have been implemented and form specially designed settings, which aim to provide a supportive environment for persons with dementia [14, 15]. Although a formal definition of SCUs is not available, they are usually located within nursing homes and provide increased safety and dementia-specific care routines [12].

Further development and differentiation could be implemented by the establishment of more innovative concepts of living environments [16–19]. These concepts have in common that the residents live in small groups in familiar settings combined with efforts to provide normal daily life [20, 21]. They provide person-centered care with an emphasis on residents’ choices, autonomy and independence [19, 22–24]. A number of studies indicate that these living arrangements can positively impact the quality of life (QoL) of residents [25].

In the last decades, a broad range of instruments to define and quantify QoL in dementia were developed, which differ widely regarding their perspective [26, 27]. According to Lawton, QoL in dementia concerns the same areas as that of healthy people, including cognitive function, activities of daily living (ADL), engagement in social behavior, and emotional status [28]. Statements can be found in the literature that QoL in dementia is inter alia influenced by BPSD [29], emotional status [30], ability to perform ADL [31], involvement in activities [32], and everyday decision-making involvement [33].

The demand for qualitative and affordable living and care arrangements will presumably increase in the future. The fact that fewer adult children will be available to provide care for their older parents will be a contributing factor [34]. Also, a shift from a perceived ‘duty to care’ to more individualized life plans will most likely lead to a decrease in informal care provision, inter alia among migrants [35]. There is a need for a systematic assessment of innovative forms of housing in terms of their impact on persons with dementia, to inform policy, practice and further research. Several reviews have already been carried out on newer housing concepts for persons with dementia [12, 20, 36, 37]. The present review aims to contribute to the emerging evidence. This review uses a literature-based definition to label housing types as innovative. Innovative housing concepts are included if they meet predefined criteria and included concepts are characterized based on the results of additional searches. The effects of innovative housing concepts on residents’ QoL, ADL, BPSD, cognition, and emotional status, as well as the robustness of these effects, are assessed.

## Methods

In order to identify studies comparing traditional and innovative housing concepts, systematic literature searches were conducted in bibliographic databases in November 2020. In a second step, information on the included concepts was collected through additional searches in reference lists of the included articles and in Google. Reporting of this review was guided by the Preferred Reporting Items for Systematic Reviews and Meta-Analyses (PRISMA) [38]. No review protocol was published in advance.

Concepts were considered being innovative when pre-defined criteria were fulfilled, namely (i) a manageable group size of less than 15 persons in a living unit, (ii) an adapted physical environment supporting homelikeness, and (iii) specific dementia-related qualification of the staff which enhances person-centered attitudes and is based on the social model of care. These criteria were derived from a list of characteristics proposed by Palm et al. (2017) and Bergmann et al. (2020), in order to delineate the included concepts from more traditional forms of care. Concepts were defined as traditional, if they did not fulfill the requirements for innovative concepts.

### Systematic database searches

The systematic database searches were conducted in Embase, PubMed and PsycInfo. The review question was decomposed into Population, Intervention, and Outcome (PIO) and the search strategy was built using a combination of “dementia”, “housing”, “quality of life” and other keywords describing population, intervention and outcomes. The search strategy was peer-reviewed using the Peer Review of Electronic Search Strategies (PRESS) 2015 Guideline Evidence-Based Checklist [39]. The full search strategies for all three databases are available in Additional file 1. In order to identify remaining studies, bibliographies of included studies were hand-searched. Results were downloaded into the EndNote reference management program (Version X9) and duplicates were removed.

### Inclusion and exclusion criteria

Only peer-reviewed publications available in full text were included. To be included, studies had to assess residents’ QoL, ADL (including the ability to perform and involvement in activities), BPSD, cognition, and emotional status. Further, studies had to report a comparison of innovative versus more traditional living concepts for persons with medium dementia. To be included, both the innovative and the traditional concepts had to offer full time (i.e., day and night) care outside of the own home. Studies were only included if they allowed a comparison between concepts.

Editorials, non-peer-reviewed publications and publications which were not available in English or German language were excluded. Cross-sectional studies were excluded if no matching was performed or if it was not statistically controlled for differences. Further, studies were excluded if no comparison was performed, if the comparator was also an innovative concept, or if the comparator was living at home. Because of the progressive course of dementia, pre-post studies were not included. Studies were excluded if ambulatory care or respite care was defined as only comparator.

### Selection and extraction

Two researchers (CS and AN) with prior experience in literature reviews independently screened titles and abstracts. Documents considered relevant were examined in full text. Any disagreements were resolved by consulting a senior researcher (SN) with extensive knowledge of literature reviews. Characteristics of the included studies were extracted in pre-specified tables, comprising concept, study design, outcomes, assessment times, and matching procedure. Further, outcomes examined in the included studies were extracted into a pre-specified table consisting of assessment instrument, outcome, results, and statistical significance. Cross-sectional studies are reported separately in the results section.

### Additional searches

In order to provide additional information on the concepts of living and care arrangements identified in the studies which fulfilled inclusion criteria, subsequent targeted searches were performed in reference lists of the included articles and in Google. Google searches were conducted by using terms and synonyms for the different concepts and considering the search tips provided by Google. The aim was to gather information on several pre-specified characteristics including aim of the living environment, underlying care concept, number of residents, environmental factors, safety measures, meals, staffing characteristics, activities, therapies, personal freedom, and financing. These criteria were defined based on internal discussions and criteria used elsewhere [20].

### Risk of bias assessment

The Checklist for Quasi-Experimental Studies (2017 version) [40] and the Checklist for Analytical Cross-Sectional Studies (2017 version) [41] of the Joanna Briggs Institute were employed to assess risk of bias. Risk of bias assessment was performed by two persons independently and results were compared. Disagreements were resolved by consulting a third person.

## Results

**Fig. 1:**
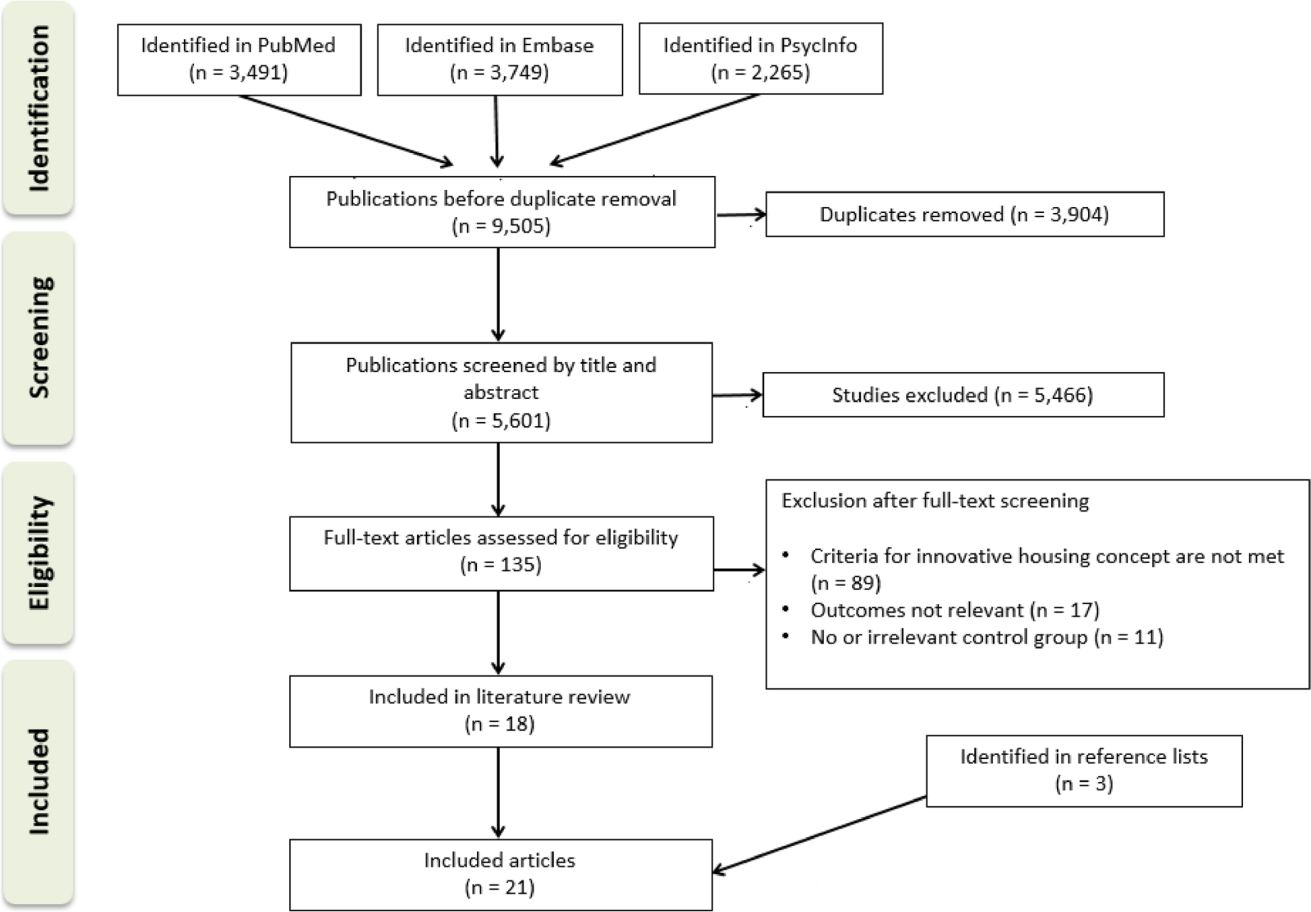
Study selection flow chart

The search was conducted in November 2020 and yielded 9,505 records (PubMed = 3,491, Embase = 3,749, PsycInfo = 2,265). A total of 3,904 duplicate records were removed electronically and manually. During the process of title and abstract screening, 5,466 records were removed because of irrelevance. The full text of 135 studies was reviewed. A list of studies which might appear to be included but which were excluded is listed in Additional file 2. Hand-searching of reference lists yielded three additional articles to be included. A total of 21 studies corresponding to 11 different concepts for living environments were included. In one of the included studies, a comparison of three living environments was undertaken, of which two environments fulfilled the pre-defined criteria for being innovative [42]. The selection process is shown in Fig. 1.

The additional targeted search for further information on the concepts of living and care arrangements identified 34 publications [7, 13, 18, 19, 22, 24, 25, 43–69].

An overview of included studies is presented in Table 1.

**Table 1:** Included studies

Concept	Study	Design (n)	Outcomes	Assessment times	Matching
Green House (USA)	Kane et al. 2007 [70]	Longitudinal study (120)	QoL, ADL, BPSD, ES	Baseline, 6 mo, 12 mo, 18 mo	No matching. Controlled for baseline characteristics
	Molony et al. 2011 [71]	Longitudinal study (25)	ADL, ES	Baseline, 1 mo, 3 mo, 6 mo	No matching. Controlled for baseline characteristics
	Yoon et al. 2015 [72]	Longitudinal study (242)	ES	Baseline, 6 mo, 12 mo, 18 mo	No matching. Controlled for baseline characteristics
	Yoon et al. 2016* [73]	Longitudinal study (242)	ADL	Baseline, 3 mo, 6 mo, 9 mo, 12 mo	No matching. Controlled for baseline characteristics
Group Living (Sweden)	Annerstedt 1994 [16]	Longitudinal study (54)	ADL, BPSD	Baseline, 6 mo, 12 mo, 36 mo	Matching according to sex, age, diagnoses and level of dementia
	Kihlgren et al. 1992 [74]	Longitudinal study (10)	ADL, BPSD, cognition, ES	Baseline, five times in 25 mo	Matching according to sex, age, level of dementia and social background
	Wimo et al. 1995 [75]	Longitudinal study (108)	ADL, BPSD, cognition	Baseline, 3 mo, 6 mo, 9 mo, 12 mo	No matching
Cantou (France)	Ritchie et al. 1992 [19]	Cross-sectional study (352)	ADL, BPSD, cognition, ES	Five times over 24 mo	Matched pairs
Group Homes (Japan)	Suzuki et al. 2008 [76]	Longitudinal study (26)	ADL, cognition	Baseline, 1 mo, 3 mo, 12 mo	Matching according to sex and type of dementia
Small-scale Group Living (Austria)	Auer et al. 2017 [21]	Longitudinal study (36)	QoL, ADL, BPSD, cognition	Baseline, 3 mo, 6 mo, 12 mo	Matched pairs
Special Care Facility (Canada)	Reimer et al. 2004 [15]	Longitudinal study (185)	ADL, BPSD, cognition, ES	Baseline, 3 mo, 6 mo, 9 mo, 12 mo	Group matching (GDS scores, age-adjusted comorbidities)
Shared-housing Arrangements (Germany)	Wolf-Ostermann et al. 2012a [23]	Longitudinal study (56)	QoL, ADL, BPSD, cognition	Baseline, 6 mo, 12 mo	No matching. Controlled for baseline characteristics
Residential Groups (Germany)	Dettbarn-Regentin et al. 2005 [77]	Longitudinal study (111)	ADL, BPSD, cognition	Baseline, 6 mo, 12 mo, 18 mo	Matching according to age, stage of dementia, mobility
Residential Care Center / Woodside Places (USA/Canada)	Warren et al. 2001 [78]	Longitudinal study (80)	ADL, BPSD, cognition, ES	Baseline, 6 mo, 12 mo, 18 mo	No matching. Controlled for baseline characteristics
Small-scale Living (Netherlands/ Belgium)	De Boer et al. 2017a*** [42]	Cross-sectional study (115)	QoL, BPSD, ES	-	Matching according to cognitive and functional status
	De Rooij et al. 2012 [79]	Longitudinal study (179)	QoL, BPSD	Baseline, 6 mo, 12 mo	No matching. Controlled for baseline characteristics
	Kok et al. 2018 [29]	Longitudinal study (115)	QoL, BPSD, cognition, ES	Baseline, 3 mo, 6 mo	No matching
	Smit et al. 2012 [14]	Cross-sectional study (1,327)	ADL	-	No matching. Controlled for baseline characteristics
	Te Boekhorst et al. 2009 [18]	Longitudinal study (164)	QoL, ADL, BPSD, cognition	Baseline, 6 mo	No matching. Controlled for baseline characteristics
	Verbeek et al. 2010 [80]	Longitudinal study (259)	QoL, BPSD	Baseline, 6 mo, 12 mo	Matched on cognitive and functional status
	Verbeek et al. 2014** [81]	Longitudinal study (259)	BPSD, ES	Baseline, 6 mo, 12 mo	Matched on cognitive and functional status
Green Care Farms (Netherlands)	De Boer et al. 2017a*** [42]	Cross-sectional study (115)	QoL, BPSD, ES	-	Matching according to cognitive and functional status

*ADL* Activities of daily living, *BPSD* Behavioral and psychological symptoms of dementia; *ES* Emotional status; *mo* Months; *QoL* Quality of life

*same population as in Yoon et al. (2015)

**same population as in Verbeek et al. (2010)

***study investigates small-scale living, Green Care farms and traditional housing

Of the 21 included studies, three studies were cross-sectional studies [19, 32, 42], while the remaining 18 studies employed a longitudinal design. Results of the risk of bias assessment for the included studies are reported in Additional file 3. In many studies, reporting quality was poor and therefore, some questions could not be answered. We found risk for selection bias and/or detection bias in several studies. For example, participants of intervention and control groups differed in important ways in some studies, inter alia regarding age, sex, BPSD, cognitive status, or ADL scores. In several studies, residents already lived in the observed facilities. This means, assessments could not be performed before the intervention/exposure in these studies, as residents had moved in before baseline measurement. Further potential bias arose by the handling of follow-ups, procedures of outcome assessments, and sample sizes.

### Characteristics of the included concepts

Detailed information on the results of the additional searches, in which further information on the included concepts of living and care arrangements were sought, is reported in Additional file 4. Table 2 gives an overview on the severity of dementia of residents, number of residents, and location of the unit.

**Table 2:** Characterization of concepts

Concept	Dementia severity	Number of residents	Location
Green House (USA)	Not stated	9–12 [13] 7–10 [22, 70] 12 or fewer [69]	On a campus or scattered in a neighborhood [22]
Group Living (Sweden)	Medium to medium-severe [43–45]	8–9 [43, 44] 6–8 [45]	Mostly in ordinary houses or apartment blocks [45]
Cantou (France)	Permanent residents, who stay until their death [19]	12–15 [19]	Separate, but in proximity to the community [19]
Group Homes (Japan)	Mild to moderate dementia [58, 59]	5–9 [59]	Sometimes in proximity or inside nursing homes [58]
Small-scale Group Living (Austria)	Dementia diagnosis [21]	10 [21]	Former casern building which also contains apartments for young families [21]
Special Care Facility (Canada)	Medium to late-stage dementia [15]	10 [64]	Separate and self-contained semi-attached bungalows [15]
Shared-housing Arrangements (Germany)	Heterogeneous residents with different care needs, residents stay until their end of life [23, 67, 68]	6–8 [23, 67, 68] Up to 12 [51]	In large apartments in residential districts, mostly in urban settings [23]
Residential Groups (Germany)	Medium to severe dementia [77]	6–12, up to 15 [77]	Small units within a larger nursing home [61]
Residential Care Center / Woodside Places (USA/Canada)	Mild to moderate dementia [24, 56]	8–12 [46, 60, 63] 8–15 [57]	Three independent households form a cluster environment shaped like the letter ‘E’ [78]
Small-scale Living (Netherlands/ Belgium)	Residents stay until their end of life [18, 32]	6–8 [32, 42, 66] 4–6 [18]	Stand-alone or at the terrain of a nursing home [32, 42]
Green Care Farms (Netherlands)	Mild, moderate and later stage dementia, residents stay until their end of life [49]	6–8 [48, 55]	On the terrain of a farm [48]

Most of the concepts are exclusively designed for residents with a diagnosis of dementia, focusing on different severities of the disease. While Group Homes and Residential Care Centers / Woodside Places target residents with mild to moderate dementia [24, 56, 58, 59], Special Care Facilities and Group Living were designed to accommodate residents with moderate to moderately-severe dementia [15, 43–45]. For Residential Groups, different concepts are possible. This includes groups with mild dementia and non-demented residents or concepts with residents suffering from moderate and severe dementia [77]. Shared-housing Arrangements are designed for heterogeneous residents with different care needs [67, 68]. Small-scale Group Living is designed for persons with care level ≥ 3 (corresponding to over 120 h of care per month) and dementia diagnosis [21]. Further information on residents’ characteristics regarding severity of disease in different innovative concepts is contained in Verbeek et al. (2009). Some concepts aim for permanent residents which can remain at the facility until death. In Cantou [19], Shared-housing Arrangements [23, 67], Small-scale Living [18, 32] and Green Care Farms [49], residents can stay until their end of life and do not have to move to a nursing home as disease progresses. Some of the Japanese Group Homes offer end-of-life care, especially if in proximity to a nursing home [53, 58]. In contrast, Group Living aims for a homogeneous group of demented. Residents should be able to communicate in a meaningful way and to get out of bed by themselves [7, 44].

Concepts also differ regarding the number of residents. While Group Living, Group Homes, Shared-housing Arragements, Small-scale Living and Green Care Farms accommodate between four and nine residents, Green Houses, Cantous, Small-scale Group Living, Special Care Facilities, Residential Groups and Woodside Places are designed to accommodate larger groups. In some concepts, residents may bring their own furniture from home [19, 21, 65]. Preparing meals on-site is part of all concepts and, in most facilities, residents are encouraged to take part in meal preparation [22, 70]. Special Care Facilities [15], Woodside Places [78] and Small-scale Living [29] also incorporate accessible garden areas, while others offer a terrace [21] or balcony [67]. While most facilities are located outside of institutions and predominantly in the community, some can be in proximity or inside traditional nursing homes, such as Group Homes [25, 65] and Residential Groups [61]. Woodside Places have been designed as a stand-alone building complex [46, 78].

Differences also exist in staffing. Nurses are employed in Group Homes [25], Residential Groups [62] and Small-scale Living [18]. In Green Houses, nursing assistants with 120 h of training are employed, which are supervised by an administrator [13, 22]. In Group Living, staff is recruited from long-term care or social home-care and led and supervised by a registered nurse, which visits the unit at least once a week [43]. Staff of Green Care Farms is often hired from regular dementia care institutions [49]. In Cantou [19] and Woodside Places [78], multipurpose staff is employed. Whereas the aforementioned concepts envisage staffing with employees which are permanently on site, some concepts rely on outpatient care services. For example, Small-scale Group Living facilities are run by a social worker for geriatric work, and care activities such as bathing or washing are carried out by ambulatory care services [21]. Similarly, in Shared-housing Arrangements care is also provided by outpatient care services [23], while this concept places an additional emphasis on the involvement of family members [23, 51, 67]

Responsibilities of staff include organizing the daily schedule and encouraging residents to take part in activities [13, 22, 43, 57, 65, 66]. Activities mainly comprise tasks of daily living, such as preparing meals, doing laundry, gardening, cleaning [15, 22, 29, 57, 76]. Small-scale Group Living facilities offer gymnastics for older adults and board games [21], Residential Groups offer a stimulating and training environment [77] and Woodside Places provide a craft program and music or dancing events [54]. However, classic activities for leisure and therapies play a subordinate role in many concepts [67, 82], while physical and mental functioning training is promoted through daily household activities or a stimulating environment [51, 76, 77].

Many descriptions mention a high degree of personal freedom. Residents can flexibly choose when to eat, be cared for, sleep or take part in activities [19, 22, 23, 57, 80]. Facilities also provide freedom in terms of decoration or spiritual needs [23, 44, 57, 65]. Financing varies and depends on country-specific legislation.

### Outcomes of the included publications

Extracted outcomes and levels of statistical significance (where reported) of the included publications are presented in Additional file 5. In the following, findings regarding QoL, ADLs, BPSD, emotional status, and cognition will be summarized.

#### QoL

Residents’ QoL was assessed in seven of the included longitudinal studies. Of these, five studies used the QUALIDEM [18, 23, 29, 79, 80], which comprises nine domains [83]. Wolf-Ostermann et al. observed an increase in mean values in Shared-housing Arrangements compared to SCUs in most domains. However, after adjusting for sex and dementia stage, statistically significant differences between Shared-housing Arrangements and SCUs could only be shown for ‘care relationship’ after 12 months [23]. In an unadjusted analysis of the mean scores aggregated over the three measurement points, de Rooij et al. found that residents in Small-scale Settings in the Netherlands had significantly higher scores in ‘social relationships’, ‘positive affect’ and ‘having something to do’. Belgian residents showed significantly higher QoL in the domain ‘negative affect’ [79]. Verbeek et al. found a higher QoL in ‘something to do’ for residents in Small-scale Living compared to residents in regular wards when scored by nursing staff and family caregivers. The domains ‘social relations’ and ‘feeling at home’ only showed significantly better QoL when scored by family caregivers. Scoring by nursing staff resulted in a lower score in ‘negative affect’ [80]. Te Boekhorst et al. observed six domains of the QUALIDEM and found significant differences in ‘having something to do’ as a result of both the univariate and multivariate regression analysis. Kok et al. found no significant differences in the QUALIDEM before and after relocation [29]. Te Boekhorst et al. also assessed QoL with the dementia QoL instrument (DQoL) [84] and found a greater sense of aesthetics in residents of Group Living than in residents of traditional nursing homes [18]. Kane et al. investigated eleven domains of QoL [85] in residents of Green Houses and two comparison sites. Residents reported better QoL than residents in one of the comparison sites in four domains (privacy, dignity, autonomy, and food enjoyment). Residents reported better QoL than residents in the other comparison site on nine measures (privacy, dignity, meaningful activity, relationship, autonomy, food enjoyment, spiritual well-being, security, and individuality) [70]. Auer et al. assessed QoL with the Quality of Life in Alzheimer's Disease measure (QoL-AD) and the QoL-AD proxy version [82] and found no significant differences between Small-scale Group Living and traditional nursing homes [21].

#### ADL

Aspects related to ADL were observed in fourteen of the included longitudinal studies. Molony et al. found increased ADL function over time in persons who had moved to the intervention sites, as measured by the minimum data set (MDS) [71]. Annerstedt used the Katz ADL index [86] and found that residents in Group Living maintained their ADL abilities better than residents in traditional institutional care after 12 months [16]. In the study by Wimo et al., the Multi-Dimensional Dementia Assessment Scale’s (MDDA) ADL subscale showed significantly less decline in Group Living residents in dressing and motor functions [75]. Suzuki et al. assessed ADLs with the Disability Assessment for Dementia (DAD) [87] and found significant improvements in the items hygiene, dressing, and eating after one, three, and 12 months in group home residents compared with before admission. Significant improvements at one or more but not all time points were observed in continence, meal preparation, telephoning, going on an outing, finance and correspondence, housework, as well as in the three subscales initiation, planning and organization, and effective performance. These improvements could not be seen in the comparison group [76]. Te Boekhorst used the Interview for the Deterioration of Daily Living activities in Dementia (IDDD) [88] and found that residents in the intervention group needed less assistance with ADL [18]. Dettbarn-Reggentin et al. used the Bartel-Index [89] and found a significant decline in the comparison group, which could not be observed in the intervention group [77]. Warren et al. assessed ADL with a combination of the Functional Assessment Measure (FAM) and the Functional Independence Measure (FIM) [90] and could not show that the rates of decline in both settings were affected by the care setting. The authors found that residents in the intervention group showed greater engagement in activities outside their private rooms compared to residents in the control group [78]. Reimer et al. used Functional Assessment Staging (FAST) [91] and found significant decline over time for all groups, while less decline was found in the intervention group when compared to the two comparison groups [15].

Auer et al. also assessed ADL with FAST and could not detect changes compared to baseline in the intervention group [21]. Kane et al. found no differences between Group Homes and the comparison sites with respect to ADLs and instrumental ADLs (IADLs) [70]. Yoon et al. retrospectively studied the effect of Green Houses on ADL function trajectory with the ADL long-form scale [92]. They found a deterioration in both groups without differences between groups when controlling for age, gender, comorbidity score, cognitive function, and depressive function [73]. In their study including ten subjects, Kihlgren et al. assessed ADL by the Katz ADL index and the MDDA ADL subscale [93] and found a deterioration in both groups [74]. Wolf-Ostermann et al. used the Barthel-Index to analyse ADL and could not prove interactions between settings and development over time [23].

#### BPSD

Fourteen of the longitudinal studies assessed BPSD. Annerstedt found a slight improvement in Group Living residents and a deterioration in control subjects on the subscale ‘symptoms common in dementia’ of the Gottfries-Bråne-Steen scale [94] in the first six months. After 12 months, no differences could be detected [16]. Kihlgren et al. used the MDDA scale [74] to assess behavioral disturbances and psychiatric symptoms in the included ten residents. Results indicated a greater deterioration in the comparison group than in the intervention group over time [74]. Wimo et al. assessed behavioral aspects with the subscale of the MDDA scale and found ‘mixing food’ to have changed favorably in the Group Living group after six months. The item ‘aggressiveness’ was significantly more frequent in Group Living residents after six and 12 months, and the items ‘care resistance’ and ‘hits patients/staff’ were more common in Group Living residents after 12 months [75]. Auer et al. used the Behavioural Pathology in Alzheimer’s Disease Frequency-Weighted Severity Scale (BEHAVE-AD-FW) scale [95] and the Empirical Behavioural Pathology in Alzheimer’s Disease Assessment Scale (E-BEHAVE-AD) scale [96] and found a significant reduction in symptoms in one control group in the BEHAVE-AD-FW [21]. In the study by Reimer et al. behavioral aspects was assessed with the Cohen Mansfield Agitation Inventory (CMAI) [97] and a decline over time in all groups was found, whereas the intervention group showed a trend for more agitated behavior from the third data collection period onward [15]. Wolf-Ostermann et al. also used the CMAI and found an increase in physical non-aggressive behavior and verbal agitation during the one-year study period in the control group (no *p*-value). In the Shared-housing Arrangement group, physical non-aggressive behavior and verbal agitation decrease slightly, while aggressive behavior significantly increases after six months’ follow-up. In addition, the Neuropsychiatric Inventory in a nursing home version (NPI-NH) [98] was used and a reduction over the one-year follow-up was found. Although Shared-housing Arrangement residents showed a more pronounced decline, no differences between groups could be shown [23]. Kane et al. analyzed the prevalence of behavioral symptoms among other quality indicators and found no statistically significant differences between groups [70]. De Rooij et al. and Verbeek et al. also used the NPI-NH and could not detect differences [79, 80]. Verbeek et al. aso assessed agitation with the CMAI and found stable total scores in the intervention group, while scores decreased in the control group. After 12 months, this resulted in a significant difference between groups [80]. In their 2014 publication, Verbeek et al. report a group effect for one domain of the NPI-NH, namely aberrant motor behavior, which was more pronounced in residents in Small-scale Living Facilities [81]. They also report a significant group by time interaction for physically non-aggressive behavior (subscale of the CMAI), with residents in Small-scale Living Facilities showing significantly more physically non-aggressive behavior than residents in traditional wards after 12 months [81]. Dettbarn-Reggentin et al. used the Nurses Observation Scale for Geriatric Patients (NOSGER) subscale to address social behavior [93]. They found a decline in the control group, whereas the intervention group significantly improved in all five subscales after 12 months, namely ‘is interested in what is going on around him / her’, ‘helps others as far as physically able’, ‘makes contact with people around’, ‘enjoys certain events’, and ‘maintains contact with friends or family’ [77]. Warren et al. assessed behavioral status with the Multidimensional Observation Scale for Elderly Subjects (MOSES) [99] and found no significant change over time in control subjects, whereas scores of the intervention group significantly increased between 12 and 18 months, indicating a deteriorating behavioral status [78]. Kok et al. used the Behavioral Observation Scale for Intramural Geriatric Psychiatry (Gedragsobservatieschaal voor Intramurale Psychogeriatrie (GIP)) [100] and found a significant difference over time in anxious behavior, with the Small-scale Homelike group showing significantly less anxious behavior [29]. Te Boekhorst et al. measured behavioral aspects with the Revised Memory and Behavior Problems Checklist (RMBPC) [101] and the Neuropsychiatric Inventory-Questionnaire (NPI-Q), which is an abridged version of the NPI [98] and found no differences between groups. Social engagement was measured with the Revised Index of Social Engagement (RISE) from the Resident Assessment Instrument (RAI) [102, 103] and it was shown that residents of Group Living Homes were significantly more socially engaged on second measurement [18].

#### Cognition

Cognition was assessed as an outcome in nine of the longitudinal studies. Suzuki et al. (2008) also used the Mini-Mental State Examination (MMSE) [104] and found no significant decrease in mean score in the intervention group after one, three or 12 months when compared to the score before admission. In the control group, mean MMSE score significantly decreased after 12 months [76]. Dettbarn-Reggentin et al. found declining MMSE scores in both groups without significant between-group differences. The intervention group showed slight improvements over time in the subscale orientation at study end [77]. Warren et al. observed a significant drop in MMSE scores after 12 months, followed by a stabilization after 18 months in the intervention group, while scores in the control group decreased over time [78]. In their analysis of ten subjects, Kihlgren et al. measured cognition over time with the MMSE and found that both groups were severely impaired at baseline. No changes were found over time in any of the groups [74].

Auer et al. found a significant decline in MMSE scores in the intervention group between first assessment and final assessment after six months, while no such decline could be found in the control groups. Brief Cognitive Rating Scale [105] (BCRS) score for short-term memory significantly decreased from three months to six months in the intervention group. In one of the control groups, scores for long-term memory and orientation significantly increased from baseline to three months. No differences over time could be found in Global Deterioration Scale (GDS) [21]. Te Boekhorst et al. found no differences between groups in MMSE scores [18]. Also, Wolf-Ostermann et al. found decreases in MMSE scores in both groups but could not detect differences between groups. Similarly, declines were found for stage of dementia, as measured with GDS [106], without significant group differences [23]. Kok et al. assessed cognition with the MMSE, GIP [100], the Information Questionnaire on Cognitive Decline in the Elderly (IQCODE) [107, 108], and various neuropsychological tests. While no differences in separate measures could be found between groups, additional analyses on domain clusters showed less decline in small scale residents three months after relocation [109]. Reimer et al. assessed cognition with the BCRS and found a significant decline over time in all groups except recent memory. For two subscales, a significant difference between groups and over time was found, with scores of the intervention group being between the scores of the two control groups [15].

#### Emotional status

Emotional status was assessed in eight of the longitudinal studies. In their analysis of ten residents, Kihlgren et al. examined depression with the Depression in Dementia scale (DD) [110] and found lower scores at assessment four compared to assessment one in the intervention group while scores increased in control group (no *p*-value reported) [111]. Reimer et al. assessed affect with the Apparent Affect Rating Scale (AARS) [112] and found less anxiety/fear and more interest in the intervention group [15]. Kane et al. assessed emotional well-being using an adapted scale previously developed and detected better emotional well-being in the intervention group compared to one of the control groups. They also found a lower prevalence of depression (but no lower prevalence of depression without antidepressants) [70]. Warren et al. assessed emotional status with the Cornell Scale for Depression (CSDD) [113] and found no significant changes over time [78]. Kok et al. used the Geriatric Depression Scale [114] (GDS-15) and found high levels of well-being at study start with no differences between groups over time [29]. Molony et al. also used the GDS-15 and found a trend toward increased scores in nursing home residents and a trend toward decreased scores in the intervention group [71]. Yoon et al. used the Mood Scale Score from the MDS and found a higher rate of increase in depressive symptoms for Group Home residents than for residents of traditional nursing homes [72]. Verbeek et al. found no difference in depressive symptoms between groups [81].

#### Cross-sectional studies

Three cross-sectional studies were included [19, 32, 42], the results of which are briefly reported. Ritchie et al. applied linear regression and found that differences between both groups in cognitive tasks and depression were independent of the type of dementia, with persons living in Cantou experiencing less symptoms. Further, significant differences in favor of Cantou were found on all five subscales of the Echelle de Comportement et Adaptation (ECA) [19] which was used to measure functional capacity. These differences were independent of the type of dementia, pathology, education, or age. Behavioral aspects observed in the study comprises aspects of mobility, verbal communication, occupation, and distressed behavior, which were measured in 27 matched pairs of residents of both groups. Cantou residents were found to be more mobile, have fewer language difficulties and engage in verbal communication more often [19].

De Boer et al. compared Green Care Farms, regular Small-scale Facilities and traditional nursing homes. They conducted a random-effects regression analysis controlling for age, gender, cognition and independence in ADL and found significant higher QoL-AD scores for residents of Green Care Farms in the proxy reports when compared to traditional nursing homes. Residents of Green Care Farms also scored higher in three domains of the QUALIDEM, namely ‘positive affect’, ‘social relations’ and ‘having something to do’. No significant differences were observed in RISE, NPI-NH, CMAI, and CSDD. In addition, no differences were observed between Small-scale Facilities and Green Care Farms [42]. Smit et al. derived data from the Living Arrangements for people with Dementia (LAD) study by randomly selecting 12 residents from each participating facility and obtaining data on QoL, activity involvement, dependency on ADL, neuropsychiatric symptoms, and demographics. They found that residents of care facilities with characteristics of group living home care are more involved in activities (overall and preferred) than residents of facilities with fewer characteristics of group living home care [32].

## Discussion

This review sought to collate evidence on the question of how innovative small-scale living arrangements are related to residents’ QoL, ADL, BPSD, cognition, and emotional status. A total of 21 studies were included, of which 18 were longitudinal studies and three employed a cross-sectional design. Some of the included studies indicate improved emotional status, activity performance, and/or social behavior, while some studies show increased BPSD in residents of innovative housing compared to more traditional settings. The effect on cognition remains indistinctive.

Regarding QoL, favorable statistically significant effects of innovative living and care arrangements on some aspects were found, including the QUALIDEM domains “having something to do” [18, 79, 80] and “social relations” [79, 80]. In the cross-sectional study from de Boer, residents also scored higher in these two domains [42]. Engagement in activities and having social contacts are both factors positively influencing QoL [42, 79, 115]. The reasons for the favorable results in these subscales remain speculative, but might be attributable to constant staffing, altered staff roles and stimulating design adaptations, which may have a positive effect on well-being and preservation of social behavior [77]. Thus, tailored activities based on the remaining capabilities of the residents which are integrated into normal daily living might explain the observed differences in these domains [42]. Of note, the Dutch study by Kok et al. found low baseline scores in the QUALIDEM domain “having something to do”, which remained low [29]. This observation could presumably be explained by the cognitively severe impairments of the residents in both groups, as estimated with the MMSE. The results of de Rooij attributed benefits to both concepts, traditional and small-scale settings, in specific subscales of the QUALIDEM [79]. The absence of significant differences in QUALIDEM scores in other domains may be due to high QoL scores at study start, which result in further improvements not being detected because of ceiling effects [29]. Further, an overall interpretation of the QoL-measures is difficult because scoring systems of the QoL-measures used in the included studies differ. For instance, in contrast to QUALIDEM, the QOL-AD does not differentiate between different levels of dementia severity and does not contain subscales [21].

Regarding ADL, results of several studies show that residents living in an innovative small-scale living facility could better maintain their abilities [15, 16, 18, 75, 77]. In addition, results of Suzuki et al. show significant improvements in basic and instrumental ADL in residents of innovative facilities, which could not be seen in persons residing in traditional facilities. The authors attribute these findings to the fact that innovative forms of housing are based on independence and autonomy in ADL, such as dressing, washing and toileting [76]. Analogously, Smit et al. suspect the reason for the higher involvement in activities to be the increased provision of opportunities or the establishment of a better setting for performing activities such as cleaning, having a conversation, or listening to music [32]. In other studies, functional decline could not be decelerated by living in an innovative small-scale setting [21, 23, 70, 73, 78, 111]. The use of different assessment scales renders a comparison between studies difficult [78]. However, it appears that Kane et al. are correct in their assertion that fears of innovative facilities providing insufficient resident stimulation compared to traditional facilities can be allayed [70].

Results on BPSD are inconclusive, with some studies indicating positive effects of small-scale living arrangements on some aspects [16, 29, 77, 80], while other studies found more pronounced BPSD in residents of innovative concepts [21, 23, 75, 78, 81]. Wimo et al. speculate that the higher incidence of BPSD in residents in small-scale living is related to the fact that these residents were more frequently observed, thus creating more opportunity to capture behavior [75]. Wolf-Ostermann et al. suppose that care practices or staffing levels could have influenced the increase in aggressive behavior [23]. Also, inherent characteristics of the innovative concept could be the reason for increased stress levels, including the fact that a group of demented persons who do not know each other live together in a spatially limited area and are cared for around the clock [75]. Contrary to these assumptions, other studies found a lower manifestation of anxiety in small-scaled living environments which, according to the authors, may be related to the homelike features, including the social and physical home environment and aspects of safety [15, 29, 70]. Optimal stimulation levels seem to be variable, with some residents feeling more comfortable in the denser and more structured traditional nursing homes, while others exhibit fewer BPSD in more homelike environments [15]. Finally, Wolf-Ostermann et al. found a reduction of BPSD during one-year follow-up, which is in-line with observations from the literature [23, 116] and could arguably be related to acclimation of the residents. A number of studies investigated patterns of (psychotropic) drug use [15, 16, 18, 19, 42, 71, 77, 79, 81]. While most studies did not detect differences, Annerstedt found decreasing neuroleptic treatment in residents of small-scale living arrangements, while doses increased in the control group [16]. In the study by Verbeek et al., residents of small-scale living arrangements used significantly fewer psychotropic drugs at all time points when compared to controls. According to the authors, it is unclear whether these differences, which already existed at the start of the study, were due to differences in the care environment or arose during the selection of residents [81]. Considering the personal and, among other things, economic impact of BPSD [117], further research on the effect of innovative housing on behavioral aspects would be advisable.

The effect of the living environment on cognitive function remains unclear. Most studies in which cognition over time was observed indicate that the decline in cognitive performance is rarely influenced by living arrangements. Accordingly, scores in the observed groups decrease without notable differences between the groups being observed [15, 23, 75, 77]. However, Suzuki et al. found that persons residing in Group Homes could maintain cognitive functions better than control subjects [76]. Other studies found improvements in subdomains [77], favorable results for different domains after analyzing domain clusters [109] or a stabilization after 18 months in the intervention group [15]. According to the authors, the reduction in MMSE- and BCRS-scores in residents of the innovative living arrangements seen by Auer et al. can possibly be explained by the fact that the residents of the small-scale group moved in only shortly before the start of the study, whereas most residents of the comparison group had already been living in the institutions for a long time and were able to adapt to the environment [21]. However, the existence of a link between admission and cognition is questionable, as studies showed that mental status might not be substantially influenced by relocation [18, 118, 119].

While three studies indicate positive effects of innovative facilities on emotional status, including less depression [111], better emotional well-being [70], less anxiety/fear and more interest [15], Yoon et al. found a higher rate of increase of depressive symptoms over time [73]. The authors suspect a detection bias, as in small-scale settings, staff might be able to recognize the residents’ mood more easily and residents might be more comfortable to express their feelings and emotions, due to the familiarity between staff and residents [73]. However, Zimmerman et al. note that residents in Green Houses rarely participate in domestic activities while at the same time, structured activities are offered infrequently [69] which, given the benefits of social engagements, might lead to depression [73].

Due to limitations of the included studies, the results have to be considered with caution and in particular, selection and detection bias cannot be ruled out. First, a number of studies included a rather small sample size, which may have been too small to detect minor differences [23, 111]. Loss of research participants due to death or discharge may have resulted in no significant differences being observed within or between groups [78]. Second, due to ethical and practical considerations the included longitudinal studies were of quasi-experimental nature and thus prone to selection bias when compared to “classical” randomized controlled trials. If persons are free to choose the living environment, groups might differ. For example, favorable results in scores measuring QoL or ADL could have been influenced by factors such as a higher functional capacity of residents in innovative living arrangements [78, 80]. Even though studies adjusted for differences between groups, it cannot be ruled out that results were influenced by unobserved factors or different rates of decline [18]. In some studies, residents already lived in the observed facilities at study start. As in these studies, residents had moved in before baseline measurement, assessments could not be performed before the exposure of interest. Third, results could have been influenced by factors related to staff. Staff was not blind of the experimental condition and was aware of being observed, which could have facilitated a Hawthorne effect [29, 70]. Also, staff was free to choose the facility they work at and innovative housing forms might be more attractive for staff with certain characteristics, which in combination might have led to a ‘natural selection process’ [80]. Further, studies indicate a higher job satisfaction in innovative living arrangements [16, 21, 80], which seems to fade after some time [16, 21]. Thus, to assess the transferability of the study results to practice, it should be examined if and to what extent normalization of the initial enthusiasm occurs [70]. Fourth, factors related to outcome assessment might have influenced results. Scales used might lack responsiveness to detect small differences within or between groups [78]. As self-reporting by residents was difficult, outcomes were largely measured by professional staff or informal caregivers. These proxy assessments may differ from the residents’ own ratings of QoL, as they filter subjective information through the rater’s personal opinion [79, 120]. The risk of bias might increase further if initial assessment is undertaken by relatives, while follow-up assessments are made by professional caregivers [18]. There is disagreement in the literature regarding the comparability of ratings by staff and relatives [121, 122]. Last, a limitation of the included cross-sectional studies is that they could not prove a causal relationship between type of facility and outcomes.

The 21 studies included in the review examine eleven different concepts from European and North American countries as well as Japan, with these concepts being defined according to specific criteria pertaining to group size, physical environment, and staff qualification. Because of these pre-defined measures, several identified studies on related concepts have not been included in this review, e.g., due to the number of residents [123] or because no specific concept was named or described in detail [124]. Despite these uniform and literature-based criteria, the concepts included in this review vary, particularly regarding target groups, group sizes, staff qualifications, responsibilities, activities, and domestic characteristics. Further, some of the included studies are dated, resulting in limited transferability of results. Finally, measuring QoL in demented persons is a topic of debate [19]. Outcomes strongly depend on the scale used and the underlying concept of QoL [21]. The relationship of QoL with BPSD, such as anxiety, agitation and disinhibition, is intuitively understandable [125]. However, it is arguable to what extent function or cognition can be used as a proxy for QoL [125]. In addition, the validity of the MMSE, which was used in most studies assessing cognition, has been questioned [73]. In that this review uses a broad concept of QoL, some measures reported here can arguably only be considered as crude and indirect signs of well-being [19]. Furthermore, some of the studies are dated and it cannot be ruled out that the concepts presented have changed in the meantime.

One of the strengths of this review is the transparent delineation between innovative and traditional concepts, based on literature-based criteria. Further, the additional searches enabled us to gather additional information on the concepts and describe them in detail. While it would have been of interest to examine the outcomes of the included studies in light of differences in the concepts, this was not considered feasible due to the limitations of included studies mentioned above.

## Conclusion

This review found some evidence that living in smaller and more homelike environments can encourage social behavior, preserve activity performance and positively affect emotional status in some residents for a certain time, while the decline in cognitive performance seems to be unaffected. These effects might be attributable to a stimulating design, small group sizes and altered staff roles, with an emphasis on independence, engagement in activities, and involvement of relatives [78]. On the other hand, these inherent characteristics might also be the reason for increased stress in some residents. The findings of this review indicate that innovative small-scale living might slow progression of dementia symptoms in some areas for a certain period of time. However, as the disease progresses, effects seem to abate [16, 126]. Thus, innovative facilities might be a valuable alternative and, depending on what level of care can be provided, should be part of a ‘continuum of care’ for residents with different degrees of functional abilities [78]. Due to several limitations, results have to be considered with caution and thus, the results also provide an incentive to undertake well-designed studies in order to provide decision makers with an evidence base. As nine of the 21 included studies were published before the year 2010 and four of these even before 2000, current in-depth studies are urgently needed, especially when considering the above-mentioned high relevance of care quality on residents' well-being [9]. In quasi-experimental designs, control for biases and confounding and choosing the optimal design under the given circumstances can facilitate causal interpretations [127]. In view of the methodological limitations of quasi-experimental studies, a transparent selection, sound matching, a blinded analysis of results, agreement on valid outcome measures, and a transparent reporting can contribute to results that are as valid as possible.

Finally, moving into a facility is a complex decision, which is among others influenced by care norms and cultural aspects [128]. Therefore, cultural nuances have to be considered when transferring concepts from one country to another [129]. Future research should also determine what preferences different population groups have in the event of dementia [49].

## Supplementary Information

**Additional file 1.** Databases and search terms used to identify relevant literature.

**Additional file 2.** List of excluded studies.

**Additional file 3.** Risk of bias assessment.

**Additional file 4.** Characteristics of the included concepts.

**Additional file 5.** Outcomes of included studies.

## Data Availability

Research data is available from the corresponding author on reasonable request.

## Abbreviations

AARS: Apparent Affect Rating Scale
ADL: Activities of daily living
BCRS: Brief Cognitive Rating Scale
BEHAVE-AD-FW: Behavioural Pathology in Alzheimer’s Disease Frequency-Weighted Severity Scale
BPSD: Behavioral and psychological symptoms of dementia
CMAI: Cohen Mansfield Agitation Inventory
CSDD: Cornell Scale for Depression
DAD: Disability Assessment for Dementia
DD: Depression in Dementia scale
E-BEHAVE-AD: Empirical Behavioural Pathology in Alzheimer’s Disease Assessment Scale
ECA: Echelle de Comportement et Adaptation
FAM: Functional Assessment Measure
FAST: Functional Assessment Staging
FIM: Functional Independence Measure
GDS: Global Deterioration Scale
GDS-15: Geriatric Depression Scale
GIP: Behavioral Observation Scale for Intramural Geriatric Psychiatry
IADL: Instrumental activities of daily living
IDDD: Interview for the Deterioration of Daily Living activities in Dementia
IQCODE: Information Questionnaire on Cognitive Decline in the Elderly
LAD: Living Arrangements for people with Dementia
MDDA: Multi-Dimensional Dementia Assessment Scale
MDS: Minimum data set
MMSE: Mini-Mental State Examination
MOSES: Multidimensional Observation Scale for Elderly Subjects
NPI-NH: Neuropsychiatric Inventory in a Nursing Home Version
NPI-Q: Neuropsychiatric Inventory-Questionnaire
NOSGER: Nurses Observation Scale for Geriatric Patients
PRESS: Peer Review of Electronic Search Strategies
PRISMA: Preferred Reporting Items for Systematic Reviews and Meta-Analyses
PIO: Population, Intervention, and Outcome
RAI: Resident Assessment Instrument
RISE: Revised Index of Social Engagement
RMBPC: Revised Memory and Behavior Problems Checklist
QoL: Quality of life
QoL-AD: Quality of Life in Alzheimer's Disease measure
SCU: Special care unit
