# Supplementary material for: Assessment of innovative living and care arrangements for persons with dementia: a systematic review
Source: BMC Geriatr. 2023 Aug 1;23:464. doi: 10.1186/s12877-023-04187-4 (PMC10391868; doi:10.1186/s12877-023-04187-4)
Supplement: Supplementary file 1 — Additional file 1. Databases and search terms used to identify relevant literature. [file 12877_2023_4187_MOESM1_ESM.docx]

**Additional File 1:** Databases and search terms used to identify relevant literature

| **Database** | **Search terms** | **Citations reviewed** |
| --- | --- | --- |
| **Medline** | ("Dementia"[Mesh] OR Dementia[tiab] OR "Alzheimer Disease"[Mesh] OR Alzheimer*[tiab]) | 3,491 |
| AND | (care[tiab] OR support[tiab] OR communit*[tiab] OR living[tiab] OR service[tiab] OR organized[tiab] OR residential[tiab] OR organised[tiab] OR housing[tiab] OR “housing"[Mesh] OR accommodatio*[tiab] OR relocation[tiab] OR facility[tiab] OR facilities[tiab] OR "Residential Facilities"[Mesh] OR "Residence Characteristics"[Mesh] OR “group home”[tiab]) |  |
| AND | (“quality of life”[tiab] OR "Quality of Life"[Mesh] OR hrqol[tiab] OR “health care quality”[tiab] OR “healthcare quality”[tiab] OR “quality of health care”[tiab] OR “quality of healthcare”[tiab] OR ADLs[tiab] OR cognition[tiab] OR “patient behav*”[tiab] OR BPSD OR “patient mood”[tiab] OR CSDD[tiab] OR GDS-15[tiab] OR QOL-AD[tiab] OR DQOL[tiab] OR adfacs[tiab] OR RMBBPC[tiab] OR ADRQL[tiab] OR QUALIDEM[tiab]) |  |
| Limiters | Restriction to “full text”, “humans”, and English and German language |  |
| **EMBASE** | (dementia:ab,ti,kw OR 'alzheimer disease':ab,ti,kw) | 3,749 |
| AND | (‘care‘:ab,ti,kw OR ‘support‘:ab,ti,kw OR communit*:ab,ti,kw OR ‘living‘:ab,ti,kw OR service*:ab,ti,kw OR facility:ab,ti,kw OR facilities:ab,ti,kw OR organized:ab,ti,kw OR residential:ab,ti,kw OR organised:ab,ti,kw OR housing*:ab,ti,kw OR accommodatio*:ab,ti,kw OR relocation:ab,ti,kw OR ‘small scale shared housing’:ab,ti,kw OR ‘special care facility’:ab,ti,kw OR ‘sheltered housing’:ab,ti,kw OR ‘group home’:ab,ti,kw) |  |
| AND | ('Quality of life’:ab,ti,kw OR Hrqol:ab,ti,kw OR 'health care quality':ab,ti,kw OR 'healthcare quality':ab,ti,kw OR ‘quality of health care':ab,ti,kw OR ‘quality of healthcare':ab,ti,kw OR ADLs:ab,ti,kw OR cognition:ab,ti,kw OR ‘patient behav*’:ab,ti,kw OR BPSD OR ‘patient mood’:ab,ti,kw OR CSDD:ab,ti,kw OR GDS-15:ab,ti,kw OR QOL-AD:ab,ti,kw OR DQOL:ab,ti,kw OR ADFACS:ab,ti,kw OR RMBPC:ab,ti,kw OR ADRQL:ab,ti,kw OR QUALIDEM:ab,ti,kw) |  |
| Limiters | ([article]/lim OR [article in press]/lim OR [data papers]/lim OR [review]/lim OR [short survey]/lim) AND ('clinical trial'/de OR 'clinical trial topic'/de OR 'cohort analysis'/de OR 'comparative study'/de OR 'controlled clinical trial'/de OR 'controlled study'/de OR 'cross sectional study'/de OR 'double blind procedure'/de OR 'evidence based medicine'/de OR 'evidence based practice'/de OR 'feasibility study'/de OR 'human'/de OR 'human experiment'/de OR 'intermethod comparison'/de OR 'intervention study'/de OR 'interview'/de OR 'longitudinal study'/de OR 'meta analysis'/de OR 'meta analysis topic'/de OR 'methodology'/de OR 'multicenter study'/de OR 'observational study'/de OR 'outcomes research'/de OR 'pilot study'/de OR 'practice guideline'/de OR 'prospective study'/de OR 'qualitative research'/de OR 'quality control'/de OR 'randomized controlled trial'/de OR 'randomized controlled trial topic'/de OR 'retrospective study'/de OR 'semi structured interview'/de OR 'single blind procedure'/de OR 'systematic review'/de OR 'systematic review topic'/de OR 'total quality management'/de OR 'validation process'/de OR 'validation study'/de) AND ([english]/lim OR [german]/lim) |  |
| **APA PsycInfo** | (dementia.ab,ti. or dementia.mh. or Alzheimer.ab,ti. or Alzheimer disease.mh.) | 2,265 |
| AND | (care.ab,ti. or support.ab,ti. or communit*.ab,ti. or living.ab,ti. or service.ab,ti. or organized.ab,ti. or residential.ab,ti. or organised.ab,ti. or housing.ab,ti. or housing.mh. or accommodation*.ab.ti. or relocation.ab,ti. or facility.ab,ti. or facilities.ab,ti. or residential facilities.mh. or Residence Characteristics.mh. or group home.ab,ti.) |  |
| AND | (quality of life.ab,ti. or quality of life.mh. or hrqol.ab,ti. or health care quality.ab,ti. or healthcare quality.ab,ti. or quality of health care.ab,ti. or quality of healthcare.ab,ti. or adls.ab,ti. or cognition.ab,ti.or patient behav*.ab,ti. or BPSD or patient mood.ab,ti. or csdd.ab,ti. or gds-15.ab,ti. or qol-ad.ab,ti. or dqol.ab,ti. or adfacs.ab,ti. or rmbbpc.ab,ti. OR ADRQL.ab,ti. OR QUALIDEM.ab,ti.) |  |
| Limiters | No limiters |  |
| Total records identified after database screening | | 9,505 |
| Total records after duplicates removed | | 5,601 |
