# Supplementary material for: Assessment of innovative living and care arrangements for persons with dementia: a systematic review
Source: BMC Geriatr. 2023 Aug 1;23:464. doi: 10.1186/s12877-023-04187-4 (PMC10391868; doi:10.1186/s12877-023-04187-4)
Supplement: Supplementary file 2 — Additional file 2. List of excluded studies. [file 12877_2023_4187_MOESM2_ESM.docx]

**Additional file 2**: List of excluded studies

| Citation | Reason for exclusion |
| --- | --- |
| Abrahamson K, Clark D, Perkins A, Arling G. Does Cognitive Impairment Influence Quality of Life Among Nursing Home Residents? Gerontologist. 2012 Oct; 52(5): 632–640. | A |
| Afendulis CC, Caudry DJ, O’Malley AJ, Kemper P, Grabowski DC, THRIVE Research Collaborative. Green House Adoption and Nursing Home Quality. Health Serv Res. 2016 Feb;51 Suppl 1(Suppl 1):454-74. | B |
| De Bruin S, Oostring SJ, Kuin Y, Hoefnagels ECM, Blauw YH, de Groot LCPGM, Schols JMGA. Green Care Farms Promote Activity Among Elderly People With Dementia. Journal of Housing For the Elderly. 2009;23(4). | C |
| Chafetz PK. Behavioral and cognitive outcomes of SCU care. The Journal of Aging and Mental Health. 1991;11(1):19-38. | A |
| Chappell NL, Reid RC. Dimensions of care for dementia sufferers in long-term care institutions: are they related to outcomes? J Gerontol B Psychol Sci Soc Sci. 2000 Jul;55(4):S234-44. | A |
| Dean R, Briggs K, Lindesay J. The domus philosophy: A prospective evaluation of two residential units for the elderly mentally ill. International Journal of Geriatric Psychiatry. 1993;8(10): 807–817. | C |
| Funaki Y, Kaneko F, Okamura H. Study on factors associated with changes in quality of life of demented elderly persons in group homes. Scand J Occup Ther. 2005 Mar;12(1):4-9. | C |
| Gnanamanickam ES, Dyer SM, Milte R, Liu E, Ratcliffe J, Crotty M. Clustered domestic model of residential care is associated with better consumer rated quality of care. International Journal for Quality in Health Care. 2019;31(6):419-25. | B |
| Kasai M, Meguro K, Akanuma K, Yamaguchi S. Alzheimer's disease patients institutionalized in group homes run by long-term care insurance exhibit fewer symptoms of behavioural problems as evaluated by the Behavioural Pathology in Alzheimer's Disease Rating Scale. Psychogeriatrics. 2015 Jun;15(2):102-108. | C |
| Morgan-Brown M. Comparing communal environments using the Assessment Tool for Occupation and Social Engagement: using interactive occupation and social engagement as outcome measures. Health & Social Care in the Community. 2018;27(3). | A |
| Nakanishi M, Nakashima T, Sawamura K. Quality of life of residents with dementia in a group-living situation: an approach to creating small, homelike environments in traditional nursing homes in Japan. Nihon Koshu Eisei Zasshi. 2012 Jan;59(1):3-10. | A |
| Sharkey SS, Hudak S, SD Horn, James B, Howes J. Frontline caregiver daily practices: a comparison study of traditional nursing homes and the Green House project sites. J Am Geriatr Soc. 2011 Jan;59(1):126-31. | B |
| Skea D, Lindesay J. An evaluation of two models of long-term residential care for elderly people with dementia. International Journal of Geriatric Psychiatry 1996;11(3):233-41. | A |
| Smit D, te Boekhorst S, de Lange J, Depla MFIA, Eefsting JA, Pot AM. The long-term effect of group living homes versus regular nursing homes for people with dementia on psychological distress of informal caregivers. Aging Ment Health. 2011 Jul 1;15(5):557-61. | B |
| Zimmerman S, Bowers BJ, Cohen LW et al (2016) New evidence on the green house model of nursing home care: synthesis of findings and implications for policy, practice, and research. Health Serv Res. 2016;51:475-496. | B |

A: Criteria for innovative housing concept are not met; B: Outcomes not relevant for this review; C: No or irrelevant control group
