# Supplementary material for: Assessment of innovative living and care arrangements for persons with dementia: a systematic review
Source: BMC Geriatr. 2023 Aug 1;23:464. doi: 10.1186/s12877-023-04187-4 (PMC10391868; doi:10.1186/s12877-023-04187-4)
Supplement: Supplementary file 3 — Additional file 3. Risk of bias assessment. [file 12877_2023_4187_MOESM3_ESM.docx]

**Additional file 3**: Risk of bias assessment

*Risk of bias assessment of longitudinal studies*

| Reference | Kane et al. 2007 [1] | Yoon 2015 [2] | Yoon et al. 2016 [3] | Molony et al. 2011 [4] | Annerstedt 1994 [5] | Kihlgren et al. 1992 [6] | Wimo et al. 1995 [7] | Suzuki et al. 2008 [8] | Auer et al. 2017 [9] | Reimer et al. 2004 [10] | Wolf-Ostermann et al. 2012a [11] | Dettbarn-Reggentin 2005 [12] | Warren et al. 2001 [13] | De Rooij et al. 2012 [14] | Kok et al. 2018 [15] | Te Boekhorst et al. 2009 [16] | Verbeek et al. 2010 [17] | Verbeek et al. 2014 [18] |
| --- | --- | --- | --- | --- | --- | --- | --- | --- | --- | --- | --- | --- | --- | --- | --- | --- | --- | --- |
| 1. Is it clear in the study what is the ‘cause’ and what is the ‘effect’? |  |  |  |  |  |  |  |  |  |  |  |  |  |  |  |  |  |  |
| 1. Were the participants included in any comparisons similar? |  |  |  |  |  |  |  |  |  |  |  |  |  |  |  |  |  |  |
| 1. Were the participants included in any comparisons receiving similar treatment/care, other than the exposure or intervention of interest? |  |  |  |  |  |  |  |  |  |  |  |  |  |  |  |  |  |  |
| 1. Was there a control group? |  |  |  |  |  |  |  |  |  |  |  |  |  |  |  |  |  |  |
| 1. Were there multiple measurements of the outcome both pre and post the intervention/exposure? |  |  |  |  |  |  |  |  |  |  |  |  |  |  |  |  |  |  |
| 1. Was follow up complete and if not, were differences between groups in terms of their follow up adequately described and analyzed? |  |  |  |  |  |  |  |  |  |  |  |  |  |  |  |  |  |  |
| 1. Were the outcomes of participants included in any comparisons measured in the same way? |  |  |  |  |  |  |  |  |  |  |  |  |  |  |  |  |  |  |
| 1. Were outcomes measured in a reliable way? |  |  |  |  |  |  |  |  |  |  |  |  |  |  |  |  |  |  |
| 1. a: statistical power analysis |  |  |  |  |  |  |  |  |  |  |  |  |  |  |  |  |  |  |
| 1. b: statistical analysis |  |  |  |  |  |  |  |  |  |  |  |  |  |  |  |  |  |  |

*Green: yes; yellow: unclear; red: no.*

**Additional information:**

**Question 1:** According to the Explanation for the critical appraisal tool for Quasi-Experimental Studies, the ‘cause’ has to occur in time before the explored ‘effect’. In all of the included studies, participants lived in the respective environments before follow-up measures were assessed.

**Question 2:** In a number of studies, participants differed in important ways, e.g. with regards to age, sex, behavior, cognitive status, or ADL scores.

**Question 3:** It was assumed that the participants included in any comparisons received similar treatment/care, other than the exposure or intervention of interest (see item 3 of the JBI tool) in most studies, as the intervention is constituted by the entire living environment the residents live in. However, in one study [7], patients either resided in their own homes or in a traditional nursing home and consequently, treatment/care was not regarded to be similar.

**Question 4:** A control group was included in all analyses.

**Question 5:** In a number of studies, residents already lived in the observed facilitites. As in these studies, residents had moved in before baseline measurement, assessments could not be performed before the intervention/exposure. Apart from that, no study performed multiple measurements pre the intervention and the question was answered with “yes” for all studies with at least one ‘real’ baseline-measurement.

**Question 6:** None of the studies have analyzed the impact of the loss to follow up of the results. Added sample members at follow-up have been selected randomly in some studies and might have differed in unmeasured ways. In most studies, information is incomplete and thus, the questions given in the Explanation for the critical appraisal tool cannot fully be answered.

**Question 7:** In none of the considered studies, differences in outcome measurements between groups with regard to instruments, timing, procedures, and instructions could be identified.

**Question 8:** In most studies, outcomes were measured by trained staff, while in some studies, respective information is lacking and the question was answered with “no”. In one study [16], residents were assessed by their informal caregivers at baseline and by Certified Nursing Assistants at the follow-up measurement. Since it cannot be ruled out that collection by different persons over time may have influenced the results, the question was answered with “no”.

**Question 9:** Only few studies mentioned that a statistical power calculation was performed.

*Risk of bias assessment of cross-sectional studies*

| Reference | Ritchie et al. 1992 [19] | de Boer et al. 2017 [20] | Smit et al. 2012 [21] |
| --- | --- | --- | --- |
| 1. Where the criteria for inclusion in the sample clearly defined? |  |  |  |
| 1. Were the study subjects and the settings described in detail? |  |  |  |
| 1. Was the exposure measured in a valid and reliable way? |  |  |  |
| 1. Were objective, standard criteria used for measurement |  |  |  |
| 1. Were confounding factors identified? |  |  |  |
| 1. Were strategies to deal with confounding factors stated? |  |  |  |
| 1. Were the outcomes measured in a valid and reliable way? |  |  |  |
| 1. Was appropriate statistical analysis used? |  |  |  |

*Green: yes; yellow: unclear.*

**References**

1. Kane RA, Lum TY, Cutler LJ, Degenholtz HB, Yu TC. Resident outcomes in small-house nursing homes: a longitudinal evaluation of the initial green house program. J Am Geriatr Soc. 2007 Jun;55(6):832-9.
2. Yoon, JY, Brown RL, Bowers BJ, Sharkey SS, Horn SD. Longitudinal Psychological Outcomes of the Small-scale Nursing Home Model: a Latent Growth Curve Zero-inflated Poisson Model. Int Psychogeriatr. 2015 Jun; 27(6): 1009–1016.
3. Yoon JY, Brown RL, Bowers BJ, Sharkey SS, Horn SD. The effects of the green house nursing home model on ADL function trajectory: a retrospective longitudinal study. Int J Nurs Stud. 2016 Jan;53:238-47.
4. Molony S, Evans LK, Jeon S, Rabig J, Straka LA. Trajectories of at-homeness and health in usual care and small house nursing homes. Gerontologist. 2011 Aug;51(4):504-15.
5. Annerstedt L. An attempt to determine the impact of group living care in comparison to traditional long-term care on demented elderly patients. Aging (Milano). 1994 Oct;6(5):372-80.
6. Kihlgren M, Bråne G, Karlsson I, Kuremyr D, Leissner P, Norberg A. Long-Term Influences on Demented Patients in Different Caring Milieus, a Collective Living Unit and a Nursing Home: A Descriptive Study. Dementia 1992;3:342-349.
7. Wimo A, Adolfsson R, Sandman P. Care for demented patients in different living conditions: Effects on cognitive function, ADL capacity and behavior. Scandinavian Journal of Primary Health Care. 1995;13:3,205-10.
8. Suzuki M, Kanamori ;. Yasuda M, Oshiro H. One-year follow-up study of elderly group-home residents with dementia. Am J Alzheimers Dis Other Demen. Aug-Sep 2008;23(4):334-43.
9. Auer S, Kienberger U, Pascher P, Geck M, Hoffmann B, Viereckl C, Span E. Wohngemeinschaft versus traditionelles Pflegeheim für Personen mit Demenz-Eine vergleichende Beobachtungsstudie. Pflegewissenschaft. 2017;19(3):156-165.
10. Reimer MA, Slaughter S, Donaldson C, Currie G, Eliasziw M. Special Care Facility Compared with Traditional Environments for Dementia Care: A Longitudinal Study of Quality of Life. *J Am Geriatr Soc.*2004;52:1085–1092.
11. Wolf-Ostermann K, Worch A, Fischer T, Wulff I, Gräske J. Health outcomes and quality of life of residents of shared-housing arrangements compared to residents of special care units - results of the Berlin DeWeGE-study. J Clin Nurs. 2012a;21(21-22):3047-60.
12. Dettbarn-Reggentin J. Studie zum Einfluss von Wohngruppenmilieus auf demenziell Erkrankte in stationären Einrichtungen. Zeitschrift für Gerontologie und Geriatrie 2005;38,95–100.
13. Warren S, Janzen W, Andiel-Hett C, Liu L, McKim HR, Schalm C. Innovative Dementia Care: Functional Status over Time of Persons with Alzheimer Disease in a Residential Care Centre Compared to Special Care Units. Dement Geriatr Cogn Disord, 2001;12:340–347.
14. De Rooij AHPM, Luijkx KG, Schaafsma J, Declerq AG, Emmerink PMJ, Schols JMGA. Quality of life of residents with dementia in traditional versus small-scale long-term care settings: a quasi-experimental study. Int J Nurs Stud. 2012 Aug;49(8):931-40.
15. Kok JS, Nielen MMA, Scherder EJA. Quality of life in small-scaled homelike nursing homes: an 8-month controlled trial. Health Qual Life Outcomes. 2018 Feb 27;16(1):38.
16. Te Boekhorst S, Depla MFIA, de Lange J, Pot AMP, Eefsting JA. The effects of group living homes on older people with dementia: a comparison with traditional nursing home care. Int J Geriatr Psychiatry. 2009 Sep;24(9):970-8.
17. Verbeek H, Zwakhalen SMG, van Rossum E, Ambergen T, Kempen GIJM, Hamers JPH. Dementia care redesigned: Effects of small-scale living facilities on residents, their family caregivers, and staff. J Am Med Dir Assoc. 2010 Nov;11(9):662-70.
18. Verbeek H, Zwakhalen SM, Van Rossum E, Ambergen T, Kempen GIJM, Hamers JPH. Effects of small-scale, home-like facilities in dementia care on residents’ behavior, and use of physical restraints and psychotropic drugs: a quasi-experimental study. Int Psychogeriatr. 2014 Apr;26(4):657-68.
19. Ritchie K, Ledésert B. The measurement of incapacity in the severely demented elderly: The validation of a behavioural assessment scale. Int J Geriatric Psychiat. 1992; 6, 217-266.
20. De Boer B, Hamers JPH, Zwakhalen SMG, Tan FES, Beerens HC, Verbeek H. Green Care Farms as Innovative Nursing Homes, Promoting Activities and Social Interaction for People With Dementia. J Am Med Dir Assoc. 2017b;18(1):40-46.
21. Smit D, de Lange J, Willemse B, Pot AM. The relationship between small-scale care and activity involvement of residents with dementia. Int Psychogeriatr. 2012 May;24(5):722-32.
