# Supplementary material for: Assessment of innovative living and care arrangements for persons with dementia: a systematic review
Source: BMC Geriatr. 2023 Aug 1;23:464. doi: 10.1186/s12877-023-04187-4 (PMC10391868; doi:10.1186/s12877-023-04187-4)
Supplement: Supplementary file 4 — Additional file 4. Characteristics of the included concepts. [file 12877_2023_4187_MOESM4_ESM.docx]

**youAdditional file 4:** Characteristics of the included concepts

**General characteristics**

*Aim and concept*

| **Aim of the concept** | **Care concept** | **Number of residents** |
| --- | --- | --- |
| *Green Houses (USA)* | | |
| Purposely built self-contained residence for frail older people who cannot remain in their own homes or with their families.  Model includes adapted physical environment, revised staff roles and a philosophy emphasizing a good quality of life, habilitation under normal (and not under therapeutic) circumstances [1, 2] and rejection of the idea that the residents primary live out the sick role [1]. Maximize the identification of and the respect for resident choices [1] | Holistic concept employing certified nursing assistants, which are assigned to a single group home and have broadened roles, including care, meal preparations, housekeeping, personal laundry, activities according to residents’ preferences and implementation of care plans [3, 2]  Caregiving staff and residents are expected to engage in direct personal relationships [1] | 9 to 12 persons [2]  /  7 to 10 persons [1, 3]  /  12 or fewer  [4] |
| *Group Living (Sweden)* | | |
| Alternative to nursing home care for persons with moderately to moderately-severe dementia who cannot remain in their own homes [5, 6, 7]  Optimized natural, secure, familiar and homelike setting [5] | Maintenance of normal, everyday life [7]  Homogeneous group is strived for [6].  All residents must be diagnosed with dementia [8], should be able to communicate in a meaningful way and to get out of bed by themselves [6], corresponding to Level II-IV in Berger Rating Scale and  Physical dependency maximum level E according to Katz index [5]  Care should profit from group dynamics [5] | 8 to 9 persons [5, 6]  6 to 8 persons [7] |
| *Cantou (France)* | | |
| Administratively autonomous institution for residents with dementia which places an emphasis on the prolongation of independent functioning [9]  Concept aims to lower cost of care, stimulate residents, provide encouragement for adaptive behaviour and respect the psychosocial reality. Autonomy should be respected and, if possible, developed [9] | Nonmedical, communal care [9]  Participation of families is expected at decision-making level and socially. Includes participation of family members in meals, activities and tasks such as cleaning clothes and tidying the person’s room [48]  But: permanent residents, who stay until their death. Additional medical and nursing care is provided if necessary in the terminal stages of illness [9] | 12 to 15 persons [9] |
| *Group Homes (Japan)* | | |
| To maintain and improve residual function in a home-like communal living environment [10]  Small-scale facility offering relatively non-institutional environment while often being in proximity to or inside nursing homes/greater institutions [11, 12]  Facilities accommodate older adults with mild to moderate dementia [13, 14]  (confirmed diagnosis of dementia is required [11] | Specialized in-home care services in home-like environment [15]  Aim is to establish a close and caring relationship between residents and staff [11]  Participation of residents in daily housekeeping as far as possible [13]  Some group homes offer end-of-life care [13, 15] (especially, if in proximity to nursing home) | Average number of 5 to 9 persons [14] |
| *Small-scale group living (Austria)* | | |
| To provide a home for persons with dementia which is oriented towards a normal daily routine. Environment should convey a feeling of protection and security while allowing residents to maintain their independence [16].  Residential group is designed for persons with care level ≥3 and dementia diagnosis [16]. | Care concept is not focused on medical care and follows the principle of normalization. A social care concept is focused. Care workers are seen as part of the household [16]. | 10 persons [16] |
| *Special Care Facility (Canada)* | | |
| Purpose-built bungalows offering comfortable and home-like living with more choices and more privacy than in traditional settings [17, 18]  The target population is adults with middle-to late-stage dementia [17] | Concept of normalization, physical environment and activities are arranged like a typical home [17]  Personal and direct contact [17] | 10 persons living in each bungalow [17, 18] |
| Shared-housing Arrangement (Germany) | | |
| To maintain self-determination, offering familial structures following the principle of normalization [19, 20, 21]  Heterogeneous residents with different care needs [22, 20] | Residential communities should be close to the "normal" everyday life of a (large) family, not to the necessities of care [21]  Attainment of autonomy [19]  Nursing staff members and other externals are “guests” [20]  Residents stay until their end of life and do not have to move to a nursing home as disease progresses [19, 20] | In most cases, six to eight people live together [19, 22, 20]  Up to 12 people [21] |
| *Residential Groups (Germany)* | | |
| Aim to support and promote social relationships and integration into the social environment of the residential group [23]  To provide a home-like living environment with familiarity, activity and human proximity [23]  Residents are treated in a respectful and trustful manner with focus on the daily activities, mobility, cognitive abilities and habits of the residents [23] | Concept of normalization [24] and principle of staff presence (“Präsenzkraft”) [43]  Different concepts possible, e.g. groups with non-demented and mildly ill residents (MMSE >17) or segregative concepts with residents suffering from moderate and severe dementia [23] | Beween six and 12 residents, in exceptions up to 15 residents [23] |
| *Residential Care Center / Woodside Places (USA/Canada)* | | |
| Living concept for persons with mild to moderate dementia [25, 26], who are mobile, are (at least) partially continent, and require no more than one person for assistance [27]  Physical environment aims to allow for flexibility and freedom of choice in daily activities and individuals‘ interactions and promote independence and autonomy [25]  Living and care environment is not dictated by staff routines [28]  Environment conveys that it “belongs to” the residents. For example, entrance leads directly into the residents’ living space [27] | Consistent with a social model of care which emphasizes resident independence, engagement in activities, minimum psychotropic medication and family involvement in care [29]  Primary responsibility of the direct care staff is to build close and caring relationships with residents [27] | Between eight and 12 beds per house [24, 30, 27]  Between 8 and 15 people [28] |
| *Small-scale Living (Netherlands/ Belgium)* | | |
| Segregative concept for residents with dementia [31]  Located in archetypical house where residents can stay until they die (complete substitute for regular nursing home care) [32, 33]  Aim is to enable residents to maintain their habitual lifestyle and keep up activities and hobbies as long as possible [34]  Emphasis on social relations between family, resident and caregiver [34] in a relatively small environment [32] | The staff forms a household with the residents [35, 32]  Small number of caregivers to build close relationship [34]  Staff is trained in person-centered care [36], has integrated tasks [37], and follows the concept of normalization [32] | Six to eight residents [38, 32, 37] or four to six residents [33] |
| *Green Care Farms (Netherlands)* | | |
| Combination of agricultural and care activities, aimed at involving residents in diverse activities which are incorporated into normal daily life [38, 35]  Concept differs from more traditional concepts, where activities often have an institutional character [40]  Services for mild, moderate and later stage dementia. Residents remain at the farm until death [41] | Care focuses on normalization and meaningful activities [39]  Individual vision of the farmer, leadership, and competences of other staff (e.g. creativity, empathy, flexibility) determine the features of care in terms of how activities and care environment are integrated [41] | usually six to eight persons [39, 35] |

*Physical environment*

| **Availability of single rooms** | **Dementia-specific environment** | **Demarcation from other units** | **Safety measures** | **other (e.g. brightness, space)** |
| --- | --- | --- | --- | --- |
| *Green Houses (USA)* | | | | |
| Each person has a private room and a full bathroom [2, 1] | Dementia-friendly small living unit without long hallways, institutional furnishing, nurses’ station or medication carts [1, 3] | Green Houses are grouped together, either on a campus or scattered in a neighborhood and blends architecturally with other homes [1]  Open relationship is cultivated with the surrounding community, visitors might engage with residents and staff in informal ways [1] | Wireless call systems and pagers [1] | Home-like design with large great room with fireplace, communal dining table, and walk-in kitchen which is open to dining room and great room, porch, laundry area [1, 2]  Embrace “smart house” technology and technical devices e.g. to support contact with a remote family [1] |
| *Group Living (Sweden)* | | | | |
| Persons with dementia live in their own flat with common areas for meals and social activities [42]  Personal flat consists of combined living and bedroom with full bathroom [5] | Adapted, flexible and homelike housing design based on residents needs which provides stimulation and the possibility for an interactive and therapeutic atmosphere [5] | Mostly located outside of institutions in ordinary houses or apartment blocks [7] | Physical environment is designed to be home-like, small and safe [6]. Combination of individual and common spaces eliminates security risks [5] | The common area consists of a kitchen, living room, dining room and a laundry room [5] |
| Cantou (France) | | | | |
| Single rooms are available around a large common kitchen and living area [9] | Individual rooms and community space with large kitchen and living room [9] | Community living in a separate area in proximity to the community [9] | Institution is an enclosed area [9] | Residents are encouraged to bring their own  furniture [9] |
| *Group Homes (Japan)* | | | | |
| Each person has private room with bed, closet, and enough space for private furniture and a television [11] | Residents are required to bring own furniture from home [11] | Sometimes in proximity or inside traditional nursing homes [12]  About 30% of Group Homes not affiliated with any in-home service or institution [12] |  | A large kitchen area and an open meeting area are in the center of each home, equipped with couches and tables where residents can eat or watch television [11]  Personal rooms are brightly lit and can be individually decorated [11]  Walls can be wood-paneled to create a warm atmosphere [11] |
| *Small-scale Group Living (Austria)* | | | | |
| Own room with own bathroom [16] | Residents may bring their own furniture [16] | Former casern building, which also contains apartments for young families and a kindergarten [16] |  | Each personal room has its own doorbell attached to the main gate. The homes are located in an urban environment [16] |
| *Special Care Facility (Canada)* | | | | |
| Private bedrooms with associated bathrooms [18] | Purpose-built dementia specific physical environment that is arranged like a typical home [17] | Six separate and self-contained  semi-attached bungalows [17] |  | Biodiverse environment (e.g., multigenerational, plants, live-in pets) [17] |
| Shared-housing Arrangement (Germany) | | | | |
| Private bedrooms. Kitchen, living room and also in some cases bathrooms are shared [20] | Typical flat structure with kitchen, living room and private bedrooms [19]  The rooms are furnished with personal items of the residents [20] | Situated in large apartments in residential districts, mostly in urban settings [19]  Served by at least one outpatient care service and completely disconnected from nursing homes [19]  Should be integrated into the respective social area and residents should be able to use cultural, religious and shopping opportunities [50, 21] | Ambient assisted living is sometimes used, for example electrical appliances are fitted with timers so that residents cannot use electrical appliances or the stove at night. The locking of the front door can be supported and the care service can be informed when residents get up and the light is switched on [20] | Depending on individual flat |
| *Residential Groups (Germany)* | | | | |
| Living room sizes of not less than 16 square meters for individual use, plus bathroom [43]  Double rooms only in justified exceptional cases [43] | Building was renovated to incorporate common area [44]  Living space addresses activation, communication, emotion and temporal structuring, kitchen serves as common area [23] | Small units within a larger nursing home, form an independent living unit, but are still organizationally integrated into nursing home [44] |  | Small residential units offering a familial living milieu [23]  Sufficiently equipped common rooms, short and clear routing, sufficiently bright rooms, light color design, hand-friendly materials [43] |
| *Residential Care Center / Woodside Places (USA/Canada)* | | | | |
| Single and double rooms with one private bathrooms per room [27] | Adapted physical environment with circulating paths in a secure yard, forming continuous loops [27] | Three units form a cluster environment shaped like the letter ‚E‘, with the arms of the E being three houses with the residents‘ rooms and a common living room and kitchen [29]  Each households is entirely independent [27] | Each unit has own courtyard and garden. All doors, except the front door, open to the garden and residents are allowed to enter the garden at all times [45]  Staff can look into the courtyard and has unobstrusive surveillance [45] | The upright of the E offers areas for activity shared by the three houses, e.g. a great room, an entertainment room, a horticulture room, a hair salon, a TV room and an arts room [29, 45]  The environment recognizes the difference between the public and private space. Specialized common spaces are provided outside the households. [27] |
| *Small-scale Living (Netherlands/ Belgium)* | | | | |
| Individual bedrooms [36] | Common kitchen and homelike elements [36] | May exist as stand-alone facilities (traditional group living is provided in small archetypical house-like facilities) or at the terrain of larger nursing homes; in the last years, traditional nursing homes have adapted the dementia wards into smaller units to provide group living [38, 36, 32] | Depends on the individual design | Physical environment is designed to be like an ordinary home environment as much as possible [38] |
| *Green Care Farms (Netherlands)* | | | | |
|  |  | On the terrain of a farm [46] | Potential hazards such as allergies, stumbling and injuries could arise from activities such as cooking, animal care and working with machinery. Care farms have to meet the usual regulations regarding safety as regular care environments [41] | Farms offer inviting and open environment [41] |

*Meals and personnel*

| **Possibility to prepare food** | **Responsibilities of personnel** | **Availability of personnel** | **Resident : staff ratio** |
| --- | --- | --- | --- |
| *Green Houses (USA)* | | | |
| Residential-style kitchen where meals are prepared on-site and which allows non-institutional dining experiences [1, 3] | Certified Nursing Assistants (called Shahbazim) are responsible for managing the home and work as self-managed team with coaching and supervision from an administrator (called Guide) [1, 2]  Tasks include personal care, meal preparation and service, housekeeping, activities, shopping and laundry [1, 2]  Staff receives 120 hours of training [1] | Certified Nursing Assistants are available [1]  Nurses, medical director, activity personnel, social workers, therapists, dieticians, and physicians act as a clinical support team which can be consulted and who perform care-planning functions, complete the Minimum Data Set and acts as a resource to residents and staff. They are not situated in the Green House, but visit the residents on a fixed schedule based on the treatment needs [1, 3]  Paperless record and state-of-the-art methods for Green House staff to communicate with professional staff and outside experts were contemplated [1] | The Green Houses model has a higher ratio of Certified Nursing Assistant staff to  residents than traditional skilled nursing facility units. Overall staff time (combined total of nursing and nonnursing hours per resident  day) is  slightly less in Green Houses homes. [2] |
| *Group Living (Sweden)* | | | |
| Cooking is possible [42] | Each resident has fixed contact person which helps in intimate matters, makes special efforts in the daily schedule for the resident and assists in contacting friends and relatives [5]  Staff is recruited from long-term care or social home-care and led and supervised by registered nurse [5]. Staff is trained to adapt program to patients’ biographies, present conditions and needs. Supervising nurse visits unit at least once a week, keeps patients’ care programme up to date and instructs the staff [5]. | Staff on-site at all times to provide support, guidance and help to the residents [5, 6]  Geriatrician and social worker provide monthly education and training in handling the residents` behavioral and psychosocial issues, but are not in direct contact with the residents` psychologists, occupational therapists or physiotherapists were used a`s instructor and supervisors for the staff but did not work directly with the patients. [5]  Relatives and friends are seen as resource persons and asked to take part in care planning and care [6] | Better staffed than ordinary nursing homes for the elderly [8] |
| Cantou (France) | | | |
| Meals are prepared by house mothers in the large kitchen area. Residents are encouraged to participate in preparation and cleaning up [9] | The staff is multipurpose [9] | Cantous have stable multipurpose staff [9]  Additional medical and nursing care being provided if necessary in the terminal stages of the illness [9]  The participation of families in the institutional life and care of the patients is an important aspect that may reduce the burden on professional staff and may improve family-staff relations [9] |  |
| Group Homes (Japan) | | | |
| Staff prepares meals for the residents. Residents can also prepare food on their own if they want to [11] | Only weak reliance on relatives [11]  Staff members work with the residents, doing activities such as painting, and feeding them and preparing food, if necessary [11] | Day: One nurse or care staff, three to four persons in total per unit  Night: one nurse or care staff per unit, one person per unit [10, 11]  Stable staff assignments [12]  In about 25% of Group Homes, nursing services are provided 24 hours a day [15]  Reported difficulties to find skilled workers in rural areas [11] | Mean of 12.1 staff members with few attending physicians [15]  Normally, three to four staff people are on duty during the day and one throughout the night [11]  Mean of 2.12 physicians, 5.75 nursing staff and 39.77 care working staff per 100 residents [12] |
| *Small-scale Group Living (Austria)* | | | |
| The employees prepare meals and eat themwith the residents [16] | Facility is run by a social worker for geriatric work [16]  Care activities are carried out by ambulatory care services (e.g. bathing, washing hair). [16] | No trained nurses are employed. Team consists of specialized social workers for work with the elderly,care assistants (volunteers and Civil Servants are optional) and a ambulatory care service [16] | 10 residents, 1 certified social worker, 3 specialized social workers, 3 assistants, 1 person doing voluntary social year, 1 person doing community service [16] |
| *Special Care Facility (Canada)* | | | |
| Residents can help in the kitchen [17] | Staff has enhanced knowledge and skills, responsibilities include personal care, leisure and rehabilitation activities (in contrast to an expert model of episodic therapist intervention) [17] |  | Enhanced staffing ratios compared to traditional concepts [17] |
| *Shared-housing Arrangement (Germany)* | | | |
| In a shared kitchen [19, 20] | Nursing care (outpatient care service) provides as much care as necessary, without limiting autonomy [19]  Involvement of family members, who actively participate in daily living in the SHA and act as legal representatives [19, 50, 21] | Embedded in a network consisting of outpatient care services, physicians, therapists, service providers (e.g. mobility assistance services), relatives, volunteers [20]  Large proportion of employees without at least three years of nursing training [21] | Depends on individual shared-housing arrangement [20] |
| *Residential Groups (Germany)* | | | |
| Meals are prepared together with residents [43] | Principle of staff presence requires more personal responsibility as well as more empathy for the wishes of the residents. Caregivers are specialist nurses [43]  Together with the residents, the caregiver organizes the day, i.e. takes care of household organization, keeping common rooms clean, organization of the meals. Compared to care in a standard residential care setting, the proportion of household-related services is about twice as high, accounting for about 42% of all services [43] | Organizational structures which are typical for nursing homes were replaced by familiarity and human proximity, resulting in constant staff assignments and presence of domestic caregivers [23]  Presence principle is intended to ensure that the same caregiver is always there during the day or at certain times [43] |  |
| *Residential Care Center / Woodside Places (USA/Canada)* | | | |
| Cooking is possible in the shared kitchen area [28] | Staffed with multipurpose companion workers without prior health care training. Staff is trained on the goals of the program on-site [29]  Staff is expected to adapt to the individual life-style of the residents [28]  Staff should build close, caring relationships with residents [27] | Personnel available throughout the living environment to engage in activities [27] |  |
| *Small-scale Living (Netherlands/ Belgium)* | | | |
| Meals are provided by staff together with residents and/or family caregivers [47] | Steady team of nurses performing integrated tasks comprising of personal and nursing care, domestic tasks (cooking, cleaning) and organization activities [38, 32, 47, 31]  Small staff determines daily routine together with residents and informal caregivers [33]  Staff is part of the household and encourages residents to participate in meaningful activities [37] | A small and fixed team of staff caring for residents is available [47] | Managed by one or two nurses each day [31] |
| *Green Care Farms (Netherlands)* | | | |
| Preparing food is part of the regular daily activities [46] | Staff is often hired from regular dementia care institutions. Many farmers and staff members have an education in health, pedagogy and/or social care, including registered nurses, nurse assistants, nurse aides, occupational therapists) [41]  Leadership role differs from nursing homes, as farmers are personally involved and motivated to transfer their approach of person-centeredness, creativity and dementia-care to their staff [38] | Differs. Some farms have agricultural production and for some, care is the main income source [35]  Often volunteers for assistance [41] |  |

*Activities, freedom and financing*

| **Outdoor space** | **Regular activities** | **Therapies** | **Personal freedom** | **Financing** |
| --- | --- | --- | --- | --- |
| *Green Houses (USA)* | | | | |
| Easily accessible inviting outdoor space [1, 3] | Household activities comprise planning and preparing meals, gardening, cleaning, caring for pets and doing laundry [1] | Green Houses use a range of prosthetic, educational and communication devices [1] | Residents can choose when to eat, be cared for, sleep, rest, or participate in activities [1] | Green Houses hold a nursing facility license and meet the legal facility requirements. Care is provided within Medicaid reimbursement scheme [1] |
| *Group Living (Sweden)* | | | | |
|  | Physiotherapists and occupational therapists instruct and supervise staff but do not work directly with the residents [5]  Daily activities include activation and emotional and social interaction like in an extended family [5] | Taking meals jointly with staff as part of the therapy [5] | Environment is adapted to residents‘ cultural values, traditions and way of living [6]  Each person has its own belongings in the private living area [6]  Function of staff is to support and help in coping with the social and physical environment [6] | Concept fulfills regulations for government subventions to the city and the municipal housing allowance for the resident [5]  The mode of care should be economically beneficial to the local government as compared to traditional institutional care [5]  A tenancy agreement exists for the private quarters with the residents [5] and each resident pays a share of the common living area, kitchen and laundry [6]  Model became widespread after governmental subsidies to the municipalities in Sweden [7] |
| *Cantou (France)* | | | | |
| - | Daily activities are principally centered around the tasks of daily living, e.g. participation in meal preparation [9]  Relatives are expected to participate in activities [48] | - | Times for dressing and washing activities are on the whole far more flexible in the Cantou than in the long-stay hospitals [9] | Are more economical than nursing homes [9] |
| *Group Homes (Japan)* | | | | |
| Group Homes have very different specifications [15] | Staff members are responsible for everyday activities [11]  Activities include individuals’ hobbies, laundry, cooking, and shopping [10]  Seasonal events such as sightseeing tours and festivals [10] | Physical and mental functional training through daily activities [10] | Freedom to decorate own room [11]  Example: Woman’s room contains Buddhist altar to carry out rituals [11] | Most Group Homes are privately owned [14]  Services are provided based on public long-term care policy. Residents are responsible for a 10% co-payment. In addition, residents have to pay costs for water and food. If resident has insufficient resources, the remainder is paid for by the national welfare system [11] |
| *Small-scale Group Living (Austria)* | | | | |
| Residents have a terrace facing an open atrium, which can be used for various activities [16] | Family-like organization of the daily routine, residents are allowed to choose their own activities [16] | e.g. gymnastics for the elderly and board games [16] | Concept aims to maintain personal freedom as far as possible [16]  All residents have access to all public areas [16] | Each resident is an independent tenant.  Contract with ambulatory care service [16] |
| *Special Care Facility (Canada)* | | | | |
| Bungalows have a small enclosed garden area [17] | Activities include helping in the kitchen, sweeping the floor, sitting by the fireplace, or going outside into the garden [17]  Activities are supported by staff [18] | Staff is responsible to support functional ability [18] |  | Publicly funded by regional continuing care system [17] |
| *Shared-housing Arrangement (Germany)* | | | | |
| Depends on individual shared-housing arrangement. Usually a terrace or a freely accessible balcony exists [20] | Daily household routine, structured by shared meals and activities [19, 20]  Activities that arise in the household are included in everyday life; classic activities for leisure play a subordinate role [20]  Community is based on social interaction (e.g. household chores) [19]  Residents are encouraged to participate in social life [19]  Sometimes it is possible to spend a vacation [20] | No classical occupational therapy beyond daily activities [21] | Personal habits of residents are respected, residents take over activities related to everyday life and are as actively involved as possible in shaping their daily lives [19]  Nursing staff provide as much care as necessary, without limiting the autonomy of persons with dementia. Nursing staff members are “guests” [19] | Landlords and care providers are bound by contract to each tenant individually and independently [1, 22, 21]  Freedom of choice with regard to the care/care provider must be given [20, 21]  There must be no operators [21]  Not subject to the Heimgesetz [20]  For the majority of residents care service is financed by social service providers [22] |
| *Residential Groups (Germany)* | | | | |
|  | Flexible organization of daily activities depending on the residents mobility, cognitive abilities and habits [23]  Daily routine is dominated by everyday household tasks instead of care activities [23] | Connected to nursing home [44]  Aim to slow down impairments caused by dementia (decrease in orientation, reduction in ability to communicate, …) by a stimulating and training environment [23] |  | Organizationally attached to nursing home [44] |
| *Residential Care Center / Woodside Places (USA/Canada)* | | | | |
| Secure outside garden and wandering area [29]  Large windows and sheltered porch spaces connect residents and staff with the outside [27] | A range of activities is offered including cooking, doing laundry, dining with the family, baking, sitting on a porch, and gardening [28]  Special activity rooms are available [45] | Residents’ involvement in group activities: craft program, music or dancing event [45]  Social interaction of residents: conversation or other signs of affection [45] | Residents can decide when to get up, eat, bathe, and participate in planned activities [28]  Individual care is offered with the aim to meet the resident‘s physical, psychosocial and spiritual needs [28]  Physical environment allows greater flexibility and freedom of choice in daily lives [24] | Aims to be more cost-effective than nursing home care [27] |
| *Small-scale Living (Netherlands/ Belgium)* | | | | |
| Garden is available [26] | Activities are determined by residents and their informal caregivers [38]  Residents  participate in meaningful household activities such as cleaning, preparing meals and doing laundry [26] |  | Daily schedule is organized by residents, family and staff [47] with a focus on the needs and wishes of the Resident [31] | Netherlands:  Residents have to make income-dependent Contributions. In addition, funded by government [34] governmental support brought small-scale living facilities to form a significant part (approx. 25%) of institutional care [37]  Belgium:  Residents pay a daily fee for lodging and food. In addition, partly funded by federal government and partly by regional authorities. [34] |
| *Green Care Farms (Netherlands)* | | | | |
| Free access to outdoor areas [38] | Activities stemming from regular farm tasks are continuously present and do not need to be specially organized [4]  Activities include  picking eggs, peeling potatoes, growing vegetables, getting milk at the dairy farm, gardening, cleaning stables, and feeding the animals as well as social and recreational activities [38, 39, 35, 46] | Regular exposure to outdoor stimuli (daylight, animals) [38] | Residents are free to go outside whenever they want [35] | In most cases, budget is directly transferred to an AWBZ-accredited institution.  Since introduction of personal budgets in the Netherlands in 2003, clients can opt for a personal budget to make own care decisions  [40]. |

**References**

1. Rabig J, Thomas W, Kane RA, Cutler LJ, McAlilly JD. Radical Redesign of Nursing Homes: Applying the Green House Concept in Tupelo, Mississippi. The Gerontologist 2006;46(4):533-539.
2. Sharkey SS, Hudak S, Horn SD, James B, Howes J. Frontline caregiver daily practices: a comparison study of traditional nursing homes and the Green House project sites. J Am Geriatr Soc. 2011   Jan;59(1):126-31.
3. Kane RA, Lum TY, Cutler LJ, Degenholtz HB, Yu TC. Resident outcomes in small-house nursing homes: a longitudinal evaluation of the initial green house program. J Am Geriatr Soc. 2007 Jun;55(6):832-9.
4. Zimmerman S, Cohen LW. Evidence behind The Green House and similar models of nursing home care. Aging Health. 2010; 6:717–737.
5. Annerstedt L. Development and consequences of group living in Sweden. Soc Sci Med. 1993 Dec;37(12):1529-38.
6. Annerstedt L. Group-Living Care: An Alternative for the Demented Elderly. Dement Geriatr Cogn Disord 1997;8:136-142.
7. Andrén S, Elmståhl S. Former family carers’ subjective experiences of burden. Dementia. 2002 1(2):241-54.
8. Rosendahl SP, Södermann M, Mazaheri M. Immigrants with dementia in Swedish residential care: an exploratory study of the experiences of their family members and Nursing staff. BMC Geriatr. 2016 Jan 16;16:18.
9. Ritchie K, Ledésert B. The measurement of incapacity in the severely demented elderly: The validation of a behavioural assessment scale. Int J Geriatric Psychiat. 1992; 6, 217-266.
10. Suzuki M, Kanamori M, Yasuda M, Oshiro H. One-year follow-up study of elderly group-home residents with dementia. Am J Alzheimers Dis Other Demen. Aug-Sep 2008;23(4):334-43.
11. Traphagan JW, Nagasawa T. Group homes for elders with dementia in Japan. Care Manag J. 2008;9(2):89-96.
12. Nakanishi M, Nakashima T, Sawamura K. Quality of life of residents with dementia in a group-living situation: an approach to creating small, homelike environments in traditional nursing homes in Japan. Nihon Koshu Eisei Zasshi. 2012 Jan;59(1):3-10.
13. Nakanishi M, Honda T. Processes of decision making and end-of-life care for patients with dementia in group homes in Japan. Arch Gerontol Geriatr. May-Jun 2009;48(3):296-9.
14. Onishi J, Suzuki Y, Umegaki H, Endo H, Kawamura T, Imaizumi M, Iguchi A. Behavioral, psychological and physical symptoms in group homes for older adults with dementia. International Psychogeriatrics. 2006;18(1):75–86.
15. Hirakawa Y, Masuda Y, Uemura K, Kuzuya M, Kimata T, Iguchi A. End-of-life care at group homes for patients with dementia in Japan. Findings from an analysis of policy-related differences. Arch Gerontol Geriatr. May-Jun 2006;42(3):233-45.
16. Auer S, Kienberger U, Pascher P, Geck M, Hoffmann B, Viereckl C, Span E. Wohngemeinschaft versus traditionelles Pflegeheim für Personen mit Demenz-Eine vergleichende Beobachtungsstudie. Pflegewissenschaft. 2017;19(3):156-165.
17. Reimer MA, Slaughter S, Donaldson C, Currie G, Eliasziw M. Special Care Facility Compared with Traditional Environments for Dementia Care: A Longitudinal Study of Quality of Life. J Am Geriatr Soc. 2004;52:1085–1092.
18. Slaughter S, Calkins M, Eliasziw M, Reimer M. Measuring physical and social environments in nursing homes for people with middle- to late-stage dementia. J Am Geriatr Soc. 2006 Sep;54(9):1436-41.
19. Wolf-Ostermann K, Worch A, Fischer T, Wulff I, Gräske J. Health outcomes and quality of life of residents of shared-housing arrangements compared to residents of special care units - results of the Berlin DeWeGE-study. J Clin Nurs. 2012a;21(21-22):3047-60.
20. Wolf-Ostermann K. Expertise zur Bewertung des Versorgungssettings ambulant betreuter Wohngemeinschaften unter besonderer Berücksichtigung von Personen mit eingeschränkter Alltagskompetenz. 2012b. Online: https://www.gkv-spitzenverband.de/media/dokumente/pflegeversicherung/forschung/projekte_unterseiten/expertise_ambulante_wgs/Expertise_Wolf-Ostermann_ambulant_betreute_WG.pdf accessed on May 8th, 2022.
21. Fischer T, Worch A, Nordheim J, Wulff I, Gräske J, Meye S, Wolf-Ostermann K. Ambulant betreute Wohngemeinschaften für alte, pflegebedürftige Menschen – Merkmale, Entwicklung und Einflussfaktoren. Pflege 2011;24(2):97-109.
22. Wolf-Ostermann K, Worch A. DeWeGe – Berliner Studie zur outcomebezogenen Evaluation der gesundheitlichen Versorgung von Menschen mit Demenz in ambulant betreuten Wohngemeinschaften. Online: https://www.ash-berlin.eu/fileadmin/Daten/Forschung/5_Projekte/WGQual/WgQual_DeWeGe_Studie.pdf accessed on May 8th, 2022
23. Dettbarn-Reggentin J. Studie zum Einfluss von Wohngruppenmilieus auf demenziell Erkrankte in stationären Einrichtungen. Zeitschrift für Gerontologie und Geriatrie 2005;38,95–100.
24. Radzey B, Kuhn C, Rauh J. Qualitätsbeurteilung der institutionellen Versorgung und Betreuung dementiell Erkrankter (Literatur-Expertise). Bundesministerium für Familie, Senioren, Frauen und Jugend, Stuttgart; Berlin; Köln: Kohlhammer, 2001.
25. McAllister CL, Silverman MA. Community formation and community roles among persons with Alzheimer's disease: a comparative study of experiences in a residential Alzheimer's facility and a traditional nursing home. Qual Health Res. 1999 Jan;9(1):65-85.
26. Janzen WM. Family members caring for relatives with Alzheimer disease in long-term care facilities. Thesis submitted to the Faculty of Graduate Studies and Research in partial fuifillment of the requirements for the degree of Doctor of Philosophy. Edmonton, Alberta 2000.
27. Danes S. Design for Dementia Care: A Retrospective Look at the Woodside Place Model, Journal of Housing For the Elderly. 2012;26(1):221-50.
28. Nagy J. Kitchens that help residents re-establish home. Alzheimer’s Care Quarterly 2002;3(1):74-7.
29. Warren S, Janzen W, Andiel-Hett C, Liu L, McKim HR, Schalm C. Innovative Dementia Care: Functional Status over Time of Persons with Alzheimer Disease in a Residential Care Centre Compared to Special Care Units. Dement Geriatr Cogn Disord, 2001;12:340–347.
30. Saxton J, Silverman M, Ricci E, Keane C, Deeley B. Maintenance of Mobility in Residents of an Alzheimer Special Care Facility. Int Psychogeriatr. 2005 Jan;10(2);213-224.
31. Te Boekhorst S, Depla MFIA, de Lange J, Pot AMP, Eefsting JA. The effects of group living homes on older people with dementia: a comparison with traditional nursing home care. Int J Geriatr Psychiatry. 2009 Sep;24(9):970-8.
32. Smit D, de Lange J, Willemse B, Pot AM. The relationship between small-scale care and activity involvement of residents with dementia. Int Psychogeriatr. 2012 May;24(5):722-32.
33. Te Boekhorst S, Depla MFIA, de Lange J, Pot AMP, Eefsting JA. The effects of group living homes on older people with dementia: a comparison with traditional nursing home care. Int J Geriatr Psychiatry. 2009 Sep;24(9):970-8.
34. De Rooij AHPM, Luijkx KG, Schaafsma J, Declerq AG, Emmerink PMJ, Schols JMGA. Quality of life of residents with dementia in traditional versus small-scale long-term care settings: a quasi-experimental study. Int J Nurs Stud. 2012 Aug;49(8):931-40.
35. De Boer B, Hamers JPH, Zwakhalen SMG, Tan FES, Beerens HC, Verbeek H. Green Care Farms as Innovative Nursing Homes, Promoting Activities and Social Interaction for People With Dementia. J Am Med Dir Assoc. 2017b;18(1):40-46.
36. Kok JS, Nielen MMA, Scherder EJA. Quality of life in small-scaled homelike nursing homes: an 8-month controlled trial. Health Qual Life Outcomes. 2018 Feb 27;16(1):38.
37. Verbeek H. Redesigning dementia care: An evaluation of small-scale homelike care environments. Universiteit Maastrich. 2011.
38. De Boer B, Hamers JPH, Zwakhalen SMG, Tan FES, Verbeek H. Quality of care and quality of life of people with dementia living at green care farms: a cross-sectional study. BMC Geriatr. 2017a;19;17(1):155.
39. International Psychogeriatric Association. Green Care Farms as an alternative living arrangement for people with dementia? Online: https://www.ipa-online.org/news-and-issues/green-care-farms-as-an-alternative-living-arrangement-for-people-with-dementia accessed on May 8th, 2022.
40. Hassink J, Grin J, Hulsink W. Multifunctional Agriculture Meets Health Care: Applying the Multi-Level Transition Sciences Perspective to Care Farming in the Netherlands. Sociol Ruralis 2013;53(2).
41. De Bruin SR, Pedersen I, Eriksen S, Hassink J, Vaandrager L, Patil GG. Care Farming for People with Dementia; What Can Healthcare Leaders Learn from This Innovative Care Concept? J Healthc Leadersh. 2020; 12: 11–18.
42. Faxén-Irving G, Andrén-Olsson B, af Geijerstam A, Basun H, Cederholm T. The effect of nutritional intervention in elderly subjects residing in group-living for the demented. Eur J Clin Nutr. 2002;56(3):221-227.
43. Reggentin H, Dettbarn-Reggentin J. Belastung wird durch Motivation kompensiert. Pflegezeitschrift 2004b;7:489-92.
44. Reggentin H, Dettbarn-Reggentin J. Die Möglichkeit eines Miteinanders. Pflegezeitschrift 2004a;3:181-4.
45. Hoglund JD, Dimotta S, Ledewitz, Saxton J. Long-term care design: Woodside Place--the role of environmental design in quality of life for residents with dementia. J Healthc Des. 1994;6:69-76.
46. De Boer B, Hamers JPH, Beerens HC, Zwakhalen SMG, Tan FES, Verbeek H. Living at the farm, innovative nursing home care for people with dementia – study protocol of an observational longitudinal study. BMC Geriatr.2015;15(144).
47. Verbeek H, Zwakhalen SMG, van Rossum E, Ambergen T, Kempen GIJM, Hamers JPH. Dementia care redesigned: Effects of small-scale living facilities on residents, their family caregivers, and staff. J Am Med Dir Assoc. 2010 Nov;11(9):662-70.
48. Ritchie K, Ledésert B. The measurement of incapacity in the severely demented elderly: The validation of a behavioural assessment scale. Int J Geriatric Psychiat. 1992  ; 6, 217-266.
49. Yokota O, Fujisawa Y, Takahashi J, Terada S, Ishihara T, Nakashima H, et al. Effects of group-home care on behavioral symptoms, quality of life, and psychotropic drug use in patients with frontotemporal dementia. J Am Med Dir Assoc. 2006;7(5):335-7.
50. Verbeek H, van Rossum E, Zwakhalen SMG, Kempen GIJM, Hamers JPH. Small, homelike care environments for older people with dementia: a literature review. Int Psychogeriatr. 2009 Apr;21(2):252-64.
