# Supplementary material for: Assessment of innovative living and care arrangements for persons with dementia: a systematic review
Source: BMC Geriatr. 2023 Aug 1;23:464. doi: 10.1186/s12877-023-04187-4 (PMC10391868; doi:10.1186/s12877-023-04187-4)
Supplement: Supplementary file 5 — Additional file 5. Outcomes of included studies. [file 12877_2023_4187_MOESM5_ESM.docx]

**Additional file 5:** Outcomes of included studies

| **Study** | **Instrument/Area** | **Outcome** | **Result** | **p-value** |
| --- | --- | --- | --- | --- |
| Green House (USA) | | | | |
| Kane et al. 2007 [1] | QoL  instrument  *(self-developed by Kane et al. 2003 [2])* | *Cedars (Comparison site 1)*  Comfort  Functional competence  Privacy  Dignity  Meaningful activity  Relationship  Autonomy  Food enjoyment  Spiritual well-being  Security  Individuality  *Trinity (Comparison site 2)*  Comfort  Functional competence  Privacy  Dignity  Meaningful activity  Relationship  Autonomy  Food enjoyment  Spiritual well-being  Security  Individuality | Coefficient (SE)  -0.022  -0.122 (0.18)  -0.818 (0.12)  -0.690 (0.13)  -0.261 (0.08)  -0.353 (0.11)  -0.439 (0.12)  -0.772 (0.16)  -0.266 (0.13)  -0.108 (0.05)  -0.475 (0.10)  0.06 (0.08)  -0.09 (0.19)  -0.27 (0.14)  -0.56 (0.15)  -0.07 (0.10)  -0.08 (0.13)  -0.27 (0.14)  -0.65 (0.18)  0.22 (0.14)  0.06 (0.06)  -0.16 (0.12) | 0.74  0.48  <0.001  <0.001  0.003  0.002  <0.001  <0.001  0.03  0.04  <0.001  0.44  0.62  0.05  <0.001  0.79  0.51  0.05  <0.001  0.12  0.34  0.17 |
|  | Emotional well-being | *Comparison site 1*  *Comparison site 2*  (Composite measure of 10 emotions, each measured on a scale from 1 to 4) | Coefficient  -1.82 (0.77)  -1.68 (0.89) | 0.01  0.06 |
|  | ADLs | *Comparison site 1*  *Comparison site 2*  (Five ADL items, each measured with a 0 to 3 score, summed for a theoretical range of 0 to 15) | Coefficient  0.42 (0.44)  -0.48 (0.50) | 0.34  0.34 |
|  | IADLs | *Comparison site 1*  *Comparison site 2*  (Six IADL items, each measured on a scale of 0 to 3, summed for a theoretical range of 0–18) | Coefficient  0.23 (0.48)  -0.25 (0.54) | 0.63  0.64  . |
|  | Quality Indicators  Prevalence of behavioral symptoms  Prevalence of depression  Prevalence depression without antidepressants | *Comparison site 1*  *Comparison site 2*  *Comparison site 1*  *Comparison site 2*  *Comparison site 1*  *Comparison site 2* | OR  0.51 (0.25)  1.56 (0.85)  OR  0.97 (0.39)  2.47 (1.05)  OR  0.76 (0.44)  1.72 (1.02) | 0.17  0.41  0.94  0.03  0.64  0.35 |
| Yoon et al. 2015 [3] | Depressive symptoms  (Results of multilevel ZIP regression) | Zero inflated part (zero vs non-zero)  Age  Female  ADL function  Cognitive function  Green House  Poisson part (level of symptoms)  Age  Female  ADL function  Cognitive function  Green House | -0.018 (0.017)  -0.045 (0.553)  -0.015 (0.026)  0.187 (0.217)  -1.020 (0.554)  OR (95% CI)  0.361 (0.122, 1.069)  0.005 (0.003)  0.008 (0.072)  0.000 (0.005)  -0.042 (0.038)  0.135 (0.060)  IRR (95% CI) 1.145 (1.017, 1.288) | 0.270  0.935  0.568  0.389  0.066  0.061  0.916  0.954  0.369  0.025 |
| Yoon et al. 2016 [4] | ADL | Baseline  *Group Home*  *Traditional*  3 months  *Group Home*  *Traditional*  6 months  *Group Home*  *Traditional*  9 months  *Group Home*  *Traditional*  12 months  *Group Home*  *Traditional*  15 months  *Group Home*  *Traditional*  18 months  *Group Home*  *Traditional* | 14.5 (6.7)  14.5 (7.4)  14.8 (7.0)  14.6 (7.5)  15.6 (6.9)  15.1 (7.3)  16.7 (6.7)  15.9 (7.2)  16.5 (6.7)  16.2 (6.7)  16.2 (6.1)  16.7 (6.7)  18.5 (4.4)  16.7 (7.0) | n.s.  n.s.  n.s.  n.s.  n.s.  n.s.  n.s. |
|  | Results of growth curve modeling (Facility type effects on the change in ADL function over time) | Conditional model  […]  Group home 🡪 Intercept  Group home 🡪 Slope | 0.11 (2.02)  -0.09 (0.19) | 0.958  0.637 |
|  | CPS  (covariate) | Baseline  *Group Home*  *Traditional*  3 months  *Group Home*  *Traditional*  6 months  *Group Home*  *Traditional*  9 months  *Group Home*  *Traditional*  12 months  *Group Home*  *Traditional*  15 months  *Group Home*  *Traditional*  18 months  *Group Home*  *Traditional* | 2.5 (1.0)  2.2 (1.2)  2.6 (1.1)  2.3 (1.3)  2.6 (1.1)  2.3 (1.3)  2.7 (1.4)  2.4 (1.2)  2.6 (1.3)  2.5 (1.2)  2.8 (1.2)  2.3 (1.3)  2.9 (1.3)  2.3 (1.5) | n.s.  n.s.  n.s.  n.s.  n.s.  n.s.  n.s. |
|  | MSS (covariate) | Baseline  *Group Home*  *Traditional*  3 months  *Group Home*  *Traditional*  6 months  *Group Home*  *Traditional*  9 months  *Group Home*  *Traditional*  12 months  *Group Home*  *Traditional*  15 months  *Group Home*  *Traditional*  18 months  *Group Home*  *Traditional* | 1.2 (1.9)  0.8 (1.5)  1.3 (1.9)  1.0 (1.7)  1.9 (2.3)  1.0 (1.6)  2.3 (2.3)  1.1 (1.7)  2.6 (2.4)  1.2 (1.7)  2.4 (2.1)  1.2 (1.9)  2.1 (2.1)  1.2 (1.8) | n.s.  n.s.  n.s.  n.s.  n.s.  n.s.  n.s. |
| Molony et al. 2011 [5] | MDS-ADL | *Baseline*  Small house  Usual care nursing home  *Wave 2*  Small house  Usual care nursing home  *Wave 3*  Small house  Usual care nursing home | Est. Least Square Means  25.67 (2.14)  29.05 (2.47)  18.48 (2.20)  27.74 (2.52)  16.91 (2.32)  27.55 (2.67) | 0.0383 |
|  | MMSE | Only reported in text |  |  |
| Group Living (Sweden) | | | | |
| Annerstedt 1994 [6] | GBS-scale | Intellectual functions | Better intellectual functions after 6 months  No difference after 12 months | <0.01  n.s. |
|  |  | Emotional functions | Less emotional disturbance after 6 months  No difference after 12 months | <0.001  n.s. |
|  |  | Motor functions | Less functional decline after 6 months  No difference in functional decline after 12 months | <0.001  n.s. |
|  |  | Symptoms common in dementia:  Confusion  Irritability  Anxiety  Fear – panic  Reduced mood  Restlessness | Less symptoms after 6 months  No difference after 12 months | <0.01  n.s. |
|  | Katz ADL index |  | No statistical comparison after 6 months  Higher ADL abilities after 12 months | <0.05 |
| Kihlgren et al. 1992 [7] | ADL | Katz ADL index  At T3, residents’ physical abilities had decreased in four patients in both groups.  At T4 Katz ADL index showed that the four residents in the CL group remained at the same level as T3. In the control group three remained at the same level and 2 decreased. | | No p-values reported |
|  | MDDA scale | **Spits out medicine**  T1  GL (n=5)  NH (n=5)  T3  GL (n=5)  NH (n=5)  **Constantly seeks attention of the staff**  T1  GL (n=5)  NH (n=5)  T3  GL (n=5)  NH (n=5)  **Disturbed sleep at night**  T1  GL (n=5)  NH (n=5)  T3  GL (n=5)  NH (n=5)  **Sad**  T1  GL (n=5)  NH (n=5)  T3  GL (n=5)  NH (n=5)  **Disturbed and restless**  T1  GL (n=5)  NH (n=5)  T3  GL (n=5)  NH (n=5)  **Easily annoyed**  T1  GL (n=5)  NH (n=5)  T3  GL (n=5)  NH (n=5)  **Fearful**  T1  GL (n=5)  NH (n=5)  T3  GL (n=5)  NH (n=5)  **Initiative**  T1  GL (n=5)  NH (n=5)  T3  GL (n=5)  NH (n=5)  **Seeks help**  T1  GL (n=5)  NH (n=5)  T3  GL (n=5)  NH (n=5) | 0  2  9  2  6  9  8  11  13  4  8  4  5  7  4  9  10  6  10  9  7  4  7  8  4  4  4  8  12  6  8  11  8  11  8  7 | No p-values reported |
|  | MMSE | Severe impairments seen in both groups at study start (T1) (MMSE range 0-13). No changes in follow-ups. | | No p-values reported |
|  | GBS scale  **GBS data was calculated according to four factors** identified by Nyth et al. (1992) [8] | impaired orientation and memory' showed identical scores at the start:  T1  CL  NH  T4  CL  NH  Impaired attention  T1  CL  NH  T4  CL  NH | **Motor functions** slightly better in the GL residents at t1. Residents of GL group showed lower motor scores at t4 than control residents.  **intellectual functions** slightly lower scores in the NH group than in the GL group at t1. At t4 both groups had increased scores with scores for 'impaired wakefulness’, 'absentmindedness', 'long-windedness’ being better in the GL group  4.9 (0.14)  4.3 (0.39)  5.5 (0.55)  5.6 (0.22)  3.2 (1.20)  2.4 (0.91)  3.5 (0.78)  3.6 (0.88) | No p-values reported  Significant difference in the development between the groups (p < 0.05)  p-value for the individual differences in progression between t1 and t4 concerning impaired attention was 0.051 |
|  | DD scale | Lower at T4 compared to T1 in the CL group and higher in the control group. | | No p-values reported |
| Wimo et al. 1995 [9] | Cognitive function  -> MMSE | Baseline  Group living (n=46)  Control group (n=62)  3 month  Group living (n=46)  Control group (n=62)  6 month  Group living (n=46)  Control group (n=62)  9 month  Group living (n=46)  Control group (n=62)  12 month  Group living (n=46)  Control group (n=62) | 14.0 (5)  14.9 (6)  13.3 (5)  13.7 (7)  12.0 (6)  12.3 (7)  12.3 (6)  12.6 (8)  10.6 (7)  11.0 (8) | n.s.  n.s.  n.s.  n.s.  n.s. |
|  | ADL-functions and behavior  -> MDDAS | **Changes in MDDAS(ADL)-scores**  Baseline  Group living (n=46)  Control group (n=62)  3 month  Group living (n=46)  Control group (n=62)  6 month  Group living (n=46)  Control group (n=62)  9 month  Group living (n=46)  Control group (n=62)  12 month  Group living (n=46)  Control group (n=62)  **Changes in MDDAS (behaviour)-scores**  Baseline  Group living (n=46)  Control group (n=62)  3 months  Group living (n=46)  Control group (n=62)  6 months  Group living (n=46)  Control group (n=62)  9 month  Group living (n=46)  Control group (n=62)  12 months  Group living (n=46)  Control group (n=62)  **Significant changes in items during the 12-month period**  Dress 0-6 months  Dress 3-6 months  Dress 6-9 months  Dress 0-12 months  Motor function (item is the MDDAS, but not included in the ADL-index) 0-12 months  Mixing food 0-6 months  Care resistance 0-12 months  Aggressiveness 0-6 months Aggressiveness 0-12 months  Hits patients/staff 0-12 months | 21.0 (3)  19.2 (4)  20.6 (3)  18.1 (5)  20.5 (4)  17.4 (5)  19.6 (4)  16.7 (5)  18.8 (5)  15.8 (5)  2.1 (3)  3.5 (4)  3.0 (4)  5.0 (5)  4.5 (4)  5.3 (5)  4.3 (4)  5.1 (6)  5.0 (5)  5.1 (5)  GL better than C  GL better than C  GL better than C  GL better than C  GL better than C  GL better than C  C better than GL  C better than GL  C better than GL  C better than GL | n.s.    Changes in index significant (p<0.05) between GL and C during the intervals 3-6 months and 3-9 months  ≤ 0.01  ≤ 0.05  ≤ 0.05  ≤ 0.01  ≤ 0.05  ≤ 0.05  ≤ 0.01  ≤ 0.01  ≤ 0.05  ≤ 0.01 |
| Cantou (France) | | | | |
| Ritchie et al. 1992 [10] | MMSE | Cantou  Hospital | M (SD)  9.49 (8.17)  9.66 (7.88) | n.s. |
|  | Receptive language score | Cantou  Hospital | M (SD)  4.0 (6.41)  2.5 (4.55) | <0.05 |
|  | Attentional deficits | Cantou  Hospital | 21%  26.8% | n.s. |
|  | Benton visual recall score | Cantou  Hospital | 0.38 (0.1)  0.23 (0.63) | n.s. |
|  | Remote memory deficits | Cantou  Hospital | 67%  59.2% | n.s. |
|  | Benton verbal fluency score | Cantou  Hospital | M (SD)  13.54 (5.89)  14.35 (5.99) | n.s. |
|  | Naming difficulties | Cantou  Hospital | 43.7%  38.6% | n.s. |
|  | Agnosia | Cantou  Hospital | 52.5%  51.9% | n.s. |
|  | Constructional apraxia | Cantou  Hospital | 90%  97% | n.s. |
|  | Depression scale score | Cantou  Hospital | 10.1 (6.3)  13.1 (7.6) | <0.01 |
|  | Physical independence | Cantou  Hospital | 51.7 (22.6)  35.6 (20.3) | <0.01 |
|  | Physical capacity | Cantou  Hospital | 13.9 (5.1)  8.4 (4.5) | <0.01 |
|  | Communication | Cantou  Hospital | 13.7 (6.8)  10.9 (5.9) | <0.01 |
|  | Occupation | Cantou  Hospital | 4.3 (3.7)  1.3 (1.5) | <0.01 |
|  | Sociability | Cantou  Hospital | 12.3 (3.6)  11.4 (2.8) | <0.01 |
|  | **Distressed behaviour**  t=1.3  Aggression (duration/min)  t=0.7  Crying (duration/min)  t=-0.7  Shouting (duration/min)  t=-2.6  Repetitive agitated movement (duration) | Cantou  Hospital  Cantou  Hospital  Cantou  Hospital  Cantou  Hospital | - 1. (0.4)  1. (0.0)   0.09 (4.6)  0.3 (0.7)  0.1 (0.2)  0.2 (1.0)  2.5 (5.8)  22.4 (39.2) | 0.22  0.5  0.49  0.02 |
| Group Homes (Japan) | | | | |
| Suzuki et al. 2008 [11] | MMSE | Before entering facility  Group Home Group (n = 13)  Control Group (n = 13)  1 mo after entering facility  Group Home Group (n = 13)  Control Group (n = 13)  3 mo after entering facility  Group Home Group (n = 13)  Control Group (n = 13)  12 mo after entering facility  Group Home Group (n = 13)  Control Group (n = 13) | Mean score (SD)  17.38 (4.93)  16.36 (7.68)  17.15 (4.54)  -  16.46 (5.61)  -  16.23 (5.43)  13.09 (7.52) | 0.980  0.851  0.783  0.049 |
|  | GBS | **Motor functions**  Before entering facility  Group Home Group (n = 13)  Control Group (n = 13)  1 mo after entering facility  Group Home Group (n = 13)  Control Group (n = 13)  3 mo after entering facility  Group Home Group (n = 13)  Control Group (n = 13)  12 mo after entering facility  Group Home Group (n = 13)  Control Group (n = 13)  **Intellectual**  Before entering facility  Group Home Group (n = 13)  Control Group (n = 13)  1 mo after entering facility  Group Home Group (n = 13)  Control Group (n = 13)  3 mo after entering facility  Group Home Group (n = 13)  Control Group (n = 13)  12 mo after entering facility  Group Home Group (n = 13)  Control Group (n = 13)  **Emotional function**  Before entering facility  Group Home Group (n = 13)  Control Group (n = 13)  1 mo after entering facility  Group Home Group (n = 13)  Control Group (n = 13)  3 mo after entering facility  Group Home Group (n = 13)  Control Group (n = 13)  12 mo after entering facility  Group Home Group (n = 13)  Control Group (n = 13)  **Different symptoms common in dementia**  Before entering facility  Group Home Group (n = 13)  Control Group (n = 13)  1 mo after entering facility  Group Home Group (n = 13)  Control Group (n = 13)  3 mo after entering facility  Group Home Group (n = 13)  Control Group (n = 13)  12 mo after entering facility  Group Home Group (n = 13)  Control Group (n = 13) | 4.31 (5.06)  3.73 (7.79)  3.62 (3.55)  -  4.46 (4.33)  -  6.31 (6.98)  6.00 (8.76)  14.08 (9.43)  10.09 (11.31)  15.77 (11.96)  -  18.77 (10.06)  -  20.92 (8.73)  14.09 (12.06)  2.38 (2.43)  4.73 (3.50)  2.85 (1.95)  -  2.08 (2.99)  -  1.38 (1.45)  5.46 (2.86)  3.92 (4.03)  11.38 (3.85)  3.54 (4.22)  -  3.23 (4.36)  -  3.15 (3.51)  11.50 (4.87) | 0.973  1.00  0.633  0.045  0.951  0.504  0.215  0.012  0.918  0.973  0.546  0.758  0.990  0.948  0.930  0.953 |
|  | DAD  Disabilities: self-care  activities | **Hygiene (0-7)**  Before entering facility  Group Home Group (n = 13)  Control Group (n = 13)  1 mo after entering facility  Group Home Group (n = 13)  Control Group (n = 13)  3 mo after entering facility  Group Home Group (n = 13)  Control Group (n = 13)  12 mo after entering facility  Group Home Group (n = 13)  Control Group (n = 13)  **Dressing (0-5)**  Before entering facility  Group Home Group (n = 13)  Control Group (n = 13)  1 mo after entering facility  Group Home Group (n = 13)  Control Group (n = 13)  3 mo after entering facility  Group Home Group (n = 13)  Control Group (n = 13)  12 mo after entering facility  Group Home Group (n = 13)  Control Group (n = 13)  **Continence (0-2)**  Before entering facility  Group Home Group (n = 13)  Control Group (n = 13)  1 mo after entering facility  Group Home Group (n = 13)  Control Group (n = 13)  3 mo after entering facility  Group Home Group (n = 13)  Control Group (n = 13)  12 mo after entering facility  Group Home Group (n = 13)  Control Group (n = 13)  **Eating (0-3)**  Before entering facility  Group Home Group (n = 13)  Control Group (n = 13)  1 mo after entering facility  Group Home Group (n = 13)  Control Group (n = 13)  3 mo after entering facility  Group Home Group (n = 13)  Control Group (n = 13)  12 mo after entering facility  Group Home Group (n = 13)  Control Group (n = 13) | 2.15 (2.54)  0.77 (1.96)  4.77 (2.42)  -  5.62 (2.18)  -  5.31 (2.36)  0.008 (0.28)  2.38 (2.26)  2.31 (1.89)  4.46 (0.97)  -  4.54 (1.20)  -  4.00 (1.73)  1.62 (0.77)  .1.15 (0.90)  0.92 (0.86)  1.92 (0.28)  -  1.69 (0.48)  -  1.08 (0.49)  0.69 (0.85)  1.23 (0.93)  1.77 (0.83)  2.46 (0.52)  -  4.62 (0.65)  -  3.00 (0.65)  1.62 (0.77) | 0.020  0.002  0.004  0.221  0.006  0.004  0.038  0.036  0.004  0.059  0.975  0.190  0.000  0.000  0.000  0.656 |
|  | Disabilities: instrumental activities | **Meal preparation (0-3)**  Before entering facility  Group Home Group (n = 13)  Control Group (n = 13)  1 mo after entering facility  Group Home Group (n = 13)  Control Group (n = 13)  3 mo after entering facility  Group Home Group (n = 13)  Control Group (n = 13)  12 mo after entering facility  Group Home Group (n = 13)  Control Group (n = 13)  **Telephoning (0-4)**  Before entering facility  Group Home Group (n = 13)  Control Group (n = 13)  1 mo after entering facility  Group Home Group (n = 13)  Control Group (n = 13)  3 mo after entering facility  Group Home Group (n = 13)  Control Group (n = 13)  12 mo after entering facility  Group Home Group (n = 13)  Control Group (n = 13)  **Going on an outing (0-5)**  Before entering facility  Group Home Group (n = 13)  Control Group (n = 13)  1 mo after entering facility  Group Home Group (n = 13)  Control Group (n = 13)  3 mo after entering facility  Group Home Group (n = 13)  Control Group (n = 13)  12 mo after entering facility  Group Home Group (n = 13)  Control Group (n = 13)  **Finance (0-2)**  Before entering facility  Group Home Group (n = 13)  Control Group (n = 13)  1 mo after entering facility  Group Home Group (n = 13)  Control Group (n = 13)  3 mo after entering facility  Group Home Group (n = 13)  Control Group (n = 13)  12 mo after entering facility  Group Home Group (n = 13)  Control Group (n = 13)  **Medication (0-2)**  Before entering facility  Group Home Group (n = 13)  Control Group (n = 13)  1 mo after entering facility  Group Home Group (n = 13)  Control Group (n = 13)  3 mo after entering facility  Group Home Group (n = 13)  Control Group (n = 13)  12 mo after entering facility  Group Home Group (n = 13)  Control Group (n = 13)  **Housework (0-5)**  Before entering facility  Group Home Group (n = 13)  Control Group (n = 13)  1 mo after entering facility  Group Home Group (n = 13)  Control Group (n = 13)  3 mo after entering facility  Group Home Group (n = 13)  Control Group (n = 13)  12 mo after entering facility  Group Home Group (n = 13)  Control Group (n = 13) | 1. (0.00) 2. (0.00)   0.23 (0.60)  -  0.23 (0.60)  -  1.15 (0.99)  0.00 (0.00)  0.08 (0.28)  0.15 (0.55)  0.62 (1.19)  -  1.38 (1.56)  -  1.38 (1.61)  0.08 (0.28)  0.08 (0.28)  0.08 (0.28)  0.85 (0.99)  -  0.77 (0.93)  -  1.54 (0.78)  0.00 (0.00)   1. (0.00) 2. (0.00)   0.38 (0.96)  -  0.62 (1.33)  -  1.62 (1.56)  0.00 (0.00)   1. (0.00) 2. (0.00)   0.31 (0.75)  -   1. (0.00)   -  0.31 (0.75)  0.00 (0.00)  0.38 (0.77)  0.23 (0.83)  1.46 (1.20)  -  1.08 (1.89)  -  2.46 (1.39)  0.00 (0.00) | 0.694  0.694  0.000  -  0.576  0.032  0.032  0.673  0.045  0.078  0.000  0.337  0.718  0.376  0.002  -  0.328  1.000  0.328  -  0.127  0.438  0.001  0.337 |
|  | DAD  Impairments | **Initiation (0-13)**  Before entering facility  Group Home Group (n = 13)  Control Group (n = 13)  1 mo after entering facility  Group Home Group (n = 13)  Control Group (n = 13)  3 mo after entering facility  Group Home Group (n = 13)  Control Group (n = 13)  12 mo after entering facility  Group Home Group (n = 13)  Control Group (n = 13)  **Planning and organization (0-12)**  Before entering facility  Group Home Group (n = 13)  Control Group (n = 13)  1 mo after entering facility  Group Home Group (n = 13)  Control Group (n = 13)  3 mo after entering facility  Group Home Group (n = 13)  Control Group (n = 13)  12 mo after entering facility  Group Home Group (n = 13)  Control Group (n = 13)  **Effective performance (0-14)**  Before entering facility  Group Home Group (n = 13)  Control Group (n = 13)  1 mo after entering facility  Group Home Group (n = 13)  Control Group (n = 13)  3 mo after entering facility  Group Home Group (n = 13)  Control Group (n = 13)  12 mo after entering facility  Group Home Group (n = 13)  Control Group (n = 13) | 2.923 (2.597)  2.24 (0.62)  7.385 (1.895)  -  7.615 (2.329)  -  10.462 (2.757)  1.30 (0.39)  1 (0.913)  1.42 (0.39)  3.077 (1.553)  -  4.308 (1.843)  -  6.231 (3.345)  0.78 (0.22)  3.077 (2.871)  2.24 (0.62)  6.538 (1.561)  -  6.923 (3.148)  -  6.231 (3.345)  1.13 (0.31) | 0.000  0.000  0.000  0.096  0.019  0.000  0.000  0.121  0.008  0.003  0.017  0.428 |
| Small-scale Group Living (Austria) | | | | |
| Auer et al. 2017 [12] | QoL-AD    QoL-AD proxy version | Experimental group  T1 (n=10)  T2 (n=9)  T3 (n=6)    Comparison group 1  T1 (n=7)  T2 (n=5)  T3 (n=7)    Comparison group 2  T1 (n=10)  T2 (n=13)  T3 (n=10)  Experimental group  T1 (n=10)  T2 (n=10)  T3 (n=10)    Comparison group 1  T1 (n=11)  T2 (n=9)  T3 (n=10)    Comparison group 2  T1 (n=14)  T2 (n=13)  T3 (n=13) | Mean score (SD)  32.52 (7.376)  30.67 (3.391)  33.11 (4.298)      33.23 (8.030)  34.04 (10.616)  31.12 (7.830)      35.24 (4.579)  31.13 (5.798)  34.34 (4.724)  26.80 (4.984)  27.30 (4.572)  23.00 (4.000)      28.18 (5.269)  28.44 (5.681)  22.20 (4.962)      28.28 (4.661)  28.54 (4.465)  24.31 (2.720) | Not reported. No significant differences between groups at any time point.    Significant differences between results of patient assessment and proxy assessment at some time points. |
|  | MMSE | Experimental group  T1 (n=10)  T2 (n=9)  T3 (n=6)  Comparison group 1  T1 (n=7)  T2 (n=5)  T3 (n=7)    Comparison group 2  T1 (n=10)  T2 (n=13)  T3 (n=10) | 16.5 (6.258)  14.1 (9.445)  13.4 (7.933)  14.6 (8.490)  13.4 (9.789)  15.0 (8.606)  15.9 (8.417)  16.2 (8.552)  16.2 (8.464) | p<0.05  p<0.05  Significant decline in EG from T1 to T3 (p=0.036)  No significant differences between the other groups at any time point. |
|  | BCRS  concentration  short-term memory  long-term memory  orientation | Experimental group  T1 (n=10)  T2 (n=9)  T3 (n=6)    Comparison group 1  T1 (n=7)  T2 (n=5)  T3 (n=7)    Comparison group 2  T1 (n=10)  T2 (n=13)  T3 (n=10)  Experimental group  T1 (n=10)  T2 (n=9)  T3 (n=6)  Comparison group 1  T1 (n=7)  T2 (n=5)  T3 (n=7)    Comparison group 2  T1 (n=10)  T2 (n=13)  T3 (n=10)  Experimental group  T1 (n=10)  T2 (n=9)  T3 (n=6)  Comparison group 1  T1 (n=7)  T2 (n=5)  T3 (n=7)    Comparison group 2  T1 (n=10)  T2 (n=13)  T3 (n=10)  Experimental group  T1 (n=10)  T2 (n=9)  T3 (n=6)  Comparison group 1  T1 (n=7)  T2 (n=5)  T3 (n=7)    Comparison group 2  T1 (n=10)  T2 (n=13)  T3 (n=10) | 4.6 (1.075)  5.1 (1.370)  4.8 (1.317)  5.09 (1.300)  3.3 (2.058)  4.9 (1.101)  3.8 (1.821)  4.15 (1.463)  4.23 (1.481)  5.1 (0.738)  4.7 (1.338)  5.5 (1.080)  5.36 (1.206)  4.4 (1.776)  5.4 (1.350)  4.47 (1.807)  4.46 (1.941)  4.54 (1.613)  4.7 (1.494)  4.4 (1.838)  4.7 (1.059)  5.0 (1.549)  3.7 (1.829)  4.4 (1.776)  4.13 (1.727)  3.46 (1.561)  3.77 (1.878)  4.9 (0.994)  4.6 (1.713)  4.8 (1.317)  5.27 (1.489)  3.9 (2.132)  4.9 (1.524)  3.93 (2.282)  4.39 (1.850)  4.31 (2.016) | No significant differences between groups at any time point.  p<0.05  p<0.05  Significant decline in EG from T2 to T3  (p=0.014)  p<0.05  p<0.05  Significant increase in CG 1 from T1 to T2 (p=0.031)  p<0.05  p<0.05  Significant increase in CG 1 from t1 to t2 (p=0.031) |
|  | FAST | Experimental group  T1 (n=10)  T2 (n=9)  T3 (n=6)  Comparison group 1  T1 (n=7)  T2 (n=5)  T3 (n=7)    Comparison group 2  T1 (n=10)  T2 (n=13)  T3 (n=10) | 5.64 (0.853)  5.88 (1.136)  5.98 (1.101)  5.95 (1.187)  6.1 (1.097)  6.1 (1.317)  5.53 (1.530)  5.46 (1.593)  6.19 (1.572) | p<0.05  p<0.05  Significant decline in CG 2 from t2 to t3  (p=0.001) |
|  | GDS | Experimental group  T1 (n=10)  T2 (n=9)  T3 (n=6)  Comparison group 1  T1 (n=7)  T2 (n=5)  T3 (n=7)    Comparison group 2  T1 (n=10)  T2 (n=13)  T3 (n=10) | 5.1 (0.738)  4.9 (0.876)  4.9 (0.994)  5.36 (1.027)  4.78 (0.972)  5.3 (1.059)  5.0 (1.414)  4.38 (1.660)  4.54 (1.613) | No significant differences between groups at any time point. |
|  | BEHAVE-AD-FW | Total  Experimental group  T1 (n=10)  T2 (n=9)  T3 (n=6)  Comparison group 1  T1 (n=7)  T2 (n=5)  T3 (n=7)    Comparison group 2  T1 (n=10)  T2 (n=13)  T3 (n=10)  Global  Experimental group  T1 (n=10)  T2 (n=9)  T3 (n=6)  Comparison group 1  T1 (n=7)  T2 (n=5)  T3 (n=7)    Comparison group 2  T1 (n=10)  T2 (n=13)  T3 (n=10) | 18.1 (13.404)  10.4 (16.880)  18.1 (25.757)  10.8 (14.811)  7.4 (14.041)  6.1 (6.887)  14.9 (13.362)  7.39 (6.752)  9.85 (7.614)  0.9 (1.197)  0.1 (0.316)  0.3 (0.675)  0.73 (1.272)  0.1 (0.316)  0.2 (0.422)  1.53 (1.302)  0.39 (0.506)  0.62 (0.87) | p<0.05  p<0.05  p<0.05  p<0.05  Significant reduction in CG2 in behavioral symptoms between t1 and t2. total: p=0.012, global p =0.008 |
|  | E-BEHAVE-AD | Total  Experimental group  T1 (n=10)  T2 (n=9)  T3 (n=6)  Comparison group 1  T1 (n=7)  T2 (n=5)  T3 (n=7)    Comparison group 2  T1 (n=10)  T2 (n=13)  T3 (n=10)  Global  Experimental group  T1 (n=10)  T2 (n=9)  T3 (n=6)  Comparison group 1  T1 (n=7)  T2 (n=5)  T3 (n=7)    Comparison group 2  T1 (n=10)  T2 (n=13)  T3 (n=10) | 1.5 (1.581)  1.2 (2.098)  2.0 (2.357)  1.55 (0.82)  1.7 (1.947)  1.4 (1.430)  1.53 (1.767)  0.85 (1.676)  1.77 (2.315)  0.8 (0.633)  0.4 (0.516)  1.0 (0.667)  0.91 (0.701)  0.8 (0.789)  0.7 (0.675)  0.8 (0.775)  0.23 (0.599)  0.62 (0.65) | No significant differences between groups at any time point. |
| Special Care Facility (Canada) | | | | |
| Reimer et al. 2004 [13] | Cognition | BCRS | No difference for concentration, past memory, and orientation between groups.    Functioning and self-care significantly different between groups. Results of EG between the two comparison sites. | 0.012 |
|  | ADL function | FAST | Less decline in intervention group | 0.016 |
|  | Behavior | CMAI | Trend for more agitation in the intervention group from third data collection period onward | 0.087 |
|  | Affect | AARS | Less anxiety/fear in intervention group    More interest in the intervention group    Trend for less increase in periods in which there was no apparent response to the environment in the intervention group | 0.003        0.017        0.079 |
| Shared-housing Arrangements (Germany) | | | | |
| Wolf-Ostermann et al. 2012a [14] | Barthel-Index  Estimated Barthel-score (GLM) | n=33  t1 (admission)  SHA  SCU  t2 (6-month follow-up)  SHA  SCU  t3 (12-month follow-up)  SHA  SCU  t1 (admission)  SHA (n=34)  SCU (n=22)  t2 (6-month follow-up)  SHA (n=34)  SCU (n=22)  t3 (12-month follow-up)  SHA (n=34)  SCU (n=22) | 56.8 (22.1)  49.6 (25.6)  45.0 (22.7)  38.8 (20.2)  36.5 (23.4)  36.2 (20.8)  58.6  64.8  43.7  46.3  36.2  49.8 | n.s.  n.s.  n.s.  Persons with severe cognitive impairment (MMSE < 10) had more often functional restrictions (t-test, p = 0.006)  Significant decrease of functional abilities over time [-17.5 points on average; GLM (Green- house-Geisser); p =0.015]. |
|  |  | Group differences for stage of dementia (GDS) at admission (GLM; p = 0.005). Residents with more severe dementia (GDS ≥ 6) had lower functional abilities on average. | | |
|  | MMSE | n=33  t1 (admission)  SHA  SCU  t2 (6-month follow-up)  SHA  SCU  t3 (12-month follow-up)  SHA  SCU | 15.7 (6.9)  12.4 (6.5)  13.8 (6.8)  8.4 (7.4)  10.8 (10.0)  8.7 (7.7) | n.s.  0.004  n.s. |
|  |  | Group differences by trend for gender (GLM; p = 0.079), with male residents having lower score values during the one-year study period on average. | | |
|  | GDS | n=33  % (n)  **t1**  4  SHA  SCU  5  SHA  SCU  6  SHA  SCU  7  SHA  SCU  % (n)  **t2**  4  SHA  SCU  5  SHA  SCU  6  SHA  SCU  7  SHA  SCU  % (n)  **t3**  4  SHA  SCU  5  SHA  SCU  6  SHA  SCU  7  SHA  SCU | 20.0 (4)  0.0 (0)  15.0 (3)  0.0 (0)  60.0 (12)  84.6 (11)  5.0 (1)  15.4 (2)  20.0 (4)  7.7 (1)  5.0 (1)  0.0 (0)  70.0 (14)  53.8 (7)  5.0 (1)  38.5 (5)   1. (0) 2. (0)   10.0 (2)  7.7 (1)  75.0 (15)  84.6 (11)  15.0 (3)  7.7 (1) |  |
|  | NPI-NH | Total score (n=33)  **t1**  SHA  SCU  **t2**  SHA  SCU  **t3**  SHA  SCU  Estimated NPI-NH- total score  **t1**  SHA (n=34)  SCU (n=22)  t2  SHA (n=34)  SCU (n=22)  **t3**  SHA (n=34)  SCU (n=22) | 32.8 (25.2)  32.6 (22.0)  25.4 (21.0)  31.5 (27.4)  15.8 (16.3)  23.3 (16.6)  47.3  34.1  28.7  36.3  17.4  20.5 | n.s.  n.s.  n.s.  Significant decrease in NPI-NH total score in both settings (GLM; p = 0.009) for the one-year follow-up period. |
|  | CMAI (behavioural problems) | % (n)  n=33  **t1**  Physical non-aggressive  SHA  SCU  Verbal agitation  SHA  SCU  Aggressive behavior  SHA  SCU  **t2**  Physical non-aggressive  SHA  SCU  Verbal agitation  SHA  SCU  Aggressive behavior  SHA  SCU  **t3**  Physical non-aggressive  SHA  SCU  Verbal agitation  SHA  SCU  Aggressive behavior  SHA  SCU | 35.0 (7)  46.2 (6)  50.0 (10)  30.8 (4)  0.0 (0)  30.8 (4)  40.0 (8)  46.2 (6)  50.0 (10)  53.8 (7)  5.0 (1)  30.8 (4)  30.0 (6)  53.8 (7)  40.0 (8)  61.5 (8)  25.0 (5)  30.8 (4) | n.s.  n.s.  0.017 (fishers exact test)  n.s.  n.s.  0.066 (fishers exact test)  n.s.  n.s.  n.s.  In SHA, aggressive behavior increased after six months of follow-up (MC-Chi-square test, p = 0.041). |
|  | QUALIDEM | n=33  **t1**  Care relationship  SHA  SCU  Positive affect  SHA  SCU  Negative affect  SHA  SCU  Restless tense behaviour  SHA  SCU  Positive self-image  SHA  SCU  Social relations  SHA  SCU  Social isolation  SHA  SCU  Feeling at home  SHA  SCU  Having something to do  SHA  SCU  **n=33**  **t2**  Care relationship  SHA  SCU  Positive affect  SHA  SCU  Negative affect  SHA  SCU  Restless tense behaviour  SHA  SCU  Positive self-image  SHA  SCU  Social relations  SHA  SCU  Social isolation  SHA  SCU  Feeling at home  SHA  SCU  Having something to do  SHA  SCU  **n=33**  **t3**  Care relationship  SHA  SCU  Positive affect  SHA  SCU  Negative affect  SHA  SCU  Restless tense behaviour  SHA  SCU  Positive self-image  SHA  SCU  Social relations  SHA  SCU  Social isolation  SHA  SCU  Feeling at home  SHA  SCU  Having something to do  SHA  SCU | 72.1 (23.3)  64.2 (25.6)  64.3 (24.7)  68.8 (21.7)  49.4 (28.7)  57.7 (30.6)  46.7 (34.3)  45.3 (32.9)  67.8 (28.9)  69.7 (26.3)  61.1 (22.1)  47.9 (17.5)  77.8 (20.7)  61.5 (25.9)  71.5 (19.7)  59.8 (30.9)  52.6 (32.5)  29.2 (28.8)  74.4 (18.6)  61.1 (18.9)  75.0 (23.2)  76.5 (23.3)  53.6 (25.7)  59.8 (22.2)  53.9 (32.1)  54.7 (35.0)  62.0 (23.5)  65.3 (33.8)  67.5 (22.9)  66.2 (20.7)  70.6 (24.3)  63.2 (26.6)  76.7 (18.3)  67.7 (29.7)  51.8 (34.6)  62.5 (24.8)  81.0 (14.6)  61.2 (21.5)  79.2 (20.0)  81.2 (23.5)  61.7 (23.0)  58.5 (30.0)  53.9 (36.3)  54.7 (32.9)  68.6 (28.6)  68.5 (33.8)  68.1 (18.8)  59.8 (14.9)  67.8 (22.2)  59.8 (28.8)  81.9 (20.0)  84.0 (22.6)  53.9 (33.1)  55.6 (33.6) | n.s.  n.s.  n.s.  n.s.  n.s.  0.078 (t-test)  0.055 (t-test)  n.s.  0.058.  0.095  n.s.  n.s.  n.s.  n.s.  n.s.  n.s.  n.s.  0.003  n.s.  n.s.  n.s.  n.s.  n.s.  n.s.  n.s.  n.s. |
| Residential Groups (Germany) | | | | |
| Dettbarn-Reggentin 2005 [15] | Barthel-Index |  | In EG, value decreases from t1 to t3 by 5 points (40.9 to 35.9).  In the CG, the value decreases from 35.9 (T1) to 23.9 (T3) | (Mann-Whitney-U-Test: t1: n.s., t3: p=0.039). |
|  | Mini-Mental-State-Examination (MMSE) |  | In EG, the value decreases from 10.3 (t1) to 9.9 (t3).  In the CG, the value decreases from 9.1 (T1) to 7.6 (T3) | (Mann-Whit- ney-U-Test: T1: n.s, T3: p=0.082). |
|  | Nurses Observation Scale for Geriatric Patients (NOSGER) | T1  Residential group  Control group  T2  Residential group  Control group  T3  Residential group  Control group | 15.85  18.03  15.96  18.79  15.33  19.55 | p<0.01  p<0.001  p<0.0001 |
| Residential Care Center / Woodside places (USA/Canada) | | | | |
| Warren et al. 2001 [16] | FAM+FIM | Mean score (SD)  6 months  RCC  SCU  12 months  RCC  SCU  18 months  RCC  SCU | 134.57 (13.84)  81.08 (29.21)  127.71 (21.47)  76.27 (27.10)  118.05 (26.80)  64.69 (24.35) | Both groups: FAM+FIM scores significantly decreased from t1 to t2 and t2 to t3,. |
|  | MMSE | Mean score (SD)  6 months  RCC  SCU  12 months  RCC  SCU  18 months  RCC  SCU | 15.43 (5.0)  4.50 (6.15)  12.86 (6.90)  3.92 (5.64)  13.24 (6.29)  3.12 (5.56) | RCC residents:  MMSE scores decreased from t1 to t2, and then stabilized  SCU residents:  No significant change over time |
|  | MOSES | Mean score (SD)  6 months  RCC  SCU  12 months  RCC  SCU  18 months  RCC  SCU | 69.43 (14.69)  96.65 (15.52)  70.14 (12.66)  98.73 (17.04)  78.48 (18.34)  99.0 (14.24) | RCC residents:  scores stable from t1 to t2 and significant increase from t2 to t3.  SCU residents:  No significant change over time |
|  | CSD | Mean score (SD)  6 months  RCC  SCU  12 months  RCC  SCU  18 months  RCC  SCU | 4.43 (3.75)  4.23 (92.57)  3.43 (3.47)  4.58 (3.58)  6.14 (6.81)  5.50 (3.72) | RCC residents:  No significant change over time  SCU residents:  No significant change over time |
|  | Engagement in Activities | first assessment  RCC  SCU  second assessment  RCC  SCU | active for 80% of scans  active for 65% of scans  active for 66% of scans  active for 45% of scans | Activity levels decreased in both groups at t2. RCC residents remained more active than SCU residents. |
| Small-scale Living (Netherlands/Belgium) / Green Care Farms (Netherlands) | | | | |
| De Boer et al. 2017 [17] | QoL-AD proxy | Total (N=115)  Green care farm (N=34)  Traditional nursing home (N=29)  Regular small- scale living facility (N = 52) | M= 31.7 SD= 5  M= 32.9* SD=4.5  M= 29.1* SD=4.9  M= 32.5 SD= 4.9 | * Significant difference at α = 0.05  Results of proxy-reports reached significance (p < 0.05, ES = 0.8): indicates that residents of green care farms had better QoL compared with residents of NH. |
|  | QoL-AD | Total (N=66)  Green care farm (N=21)  Traditional nursing home (N=15)  Regular small- scale living facility (N = 30) | M= 37.3 SD= 4.7  M= 37.6 SD= 4.1  M= 35.2 SD= 6  M= 38.2 SD= 4.2 | Indicates a meaningful difference (3 or more points) between residents in green care farm and NH. |
|  | QUALIDEM | Care relationship  Total (N=115)  Green care farm (N=34)  Traditional nursing home (N=29)  Regular small- scale living facility (N = 52) | M= 15.1 SD= 4.6  M= 16 SD= 4.9  M= 14.9 SD= 4.2  M= 14.6 SD= 4.6 |  |
|  |  | Positive affect  Total (N=115)  Green care farm (N=34)  Traditional nursing home (N=29)  Regular small- scale living facility (N = 52) | M= 14.1 SD= 3.7  M= 15.8* SD=3.6  M= 12.9* SD=3.5  M= 13.8 SD= 3.6 | * Significant difference at α = 0.05 compared to NH |
|  |  | Negative affect  Total (N=115)  Green care farm (N=34)  Traditional nursing home (N=29)  Regular small- scale living facility (N = 52) | M= 6 SD= 2.2  M= 6 SD= 2.6  M= 6.7 SD= 2.1  M= 5.6 SD= 2.1 |  |
|  |  | restless tense behavior  Total (N=115)  Green care farm (N=34)  Traditional nursing home (N=29)  Regular small- scale living facility (N = 52) | M= 5.4 SD= 2.9  M= 5.2 SD= 2.7  M= 5.5 SD= 2.8  M= 5.5 SD= 3 |  |
|  |  | positive self-image  Total (N=115)  Green care farm (N=34)  Traditional nursing home (N=29)  Regular small- scale living facility (N = 52) | M= 7.1 SD= 1.9  M= 7.3 SD= 2.1  M= 7.8 SD= 1.6  M= 6.6 SD= 2 |  |
|  |  | social relations  Total (N=115)  Green care farm (N=34)  Traditional nursing home (N=29)  Regular small- scale living facility (N = 52) | M= 12 SD= 3.7  M= 13* SD= 3.5  M= 10.4* SD=3.8  M= 12.3 SD= 3.6 | * Significant difference at α = 0.05 compared to NH |
|  |  | social isolation  Total (N=115)  Green care farm (N=34)  Traditional nursing home (N=29)  Regular small- scale living facility (N = 52) | M= 6.5 SD= 2.2  M= 6.9 SD= 2.4  M= 6.7 SD= 1.8  M= 6 SD= 2.3 |  |
|  |  | feeling at home  Total (N=115)  Green care farm (N=34)  Traditional nursing home (N=29)  Regular small- scale living facility (N = 52) | M= 9.6 SD= 2.5  M= 9.5 SD= 2.9  M= 9.9 SD= 2.2  M= 9.4 SD= 2.3 |  |
|  |  | having something to do  Total (N=115)  Green care farm (N=34)  Traditional nursing home (N=29)  Regular small- scale living facility (N = 52) | M= 2.7 SD= 2  M= 3* SD= 2.2  M= 1.6* SD= 1.8  M= 3 SD= 1.9 | * Significant difference at α = .05 compared to NH |
|  | RISE | Total (N=115)  Green care farm (N=34)  Traditional nursing home (N=29)  Regular small- scale living facility (N = 52) | M= 4.1 SD= 2  M= 4.4 SD= 1.9  M= 3.4 SD= 1.8  M= 4.4 SD= 2 |  |
|  | NPI-NH | Total (N=115)  Green care farm (N=34)  Traditional nursing home (N=29)  Regular small- scale living facility (N = 52) | M= 15.9 SD=15.7  M= 17.3 SD=17.5  M= 18.6 SD= 14  M= 13.6 SD=15.3 |  |
|  | CMAI | Total (N=115)  Green care farm (N=34)  Traditional nursing home (N=29)  Regular small- scale living facility (N = 52) | M= 41.9 SD=12.5  M= 41.5 SD=12.2  M= 42.4 SD=11.3  M= 41.8 SD=13.5 |  |
|  | CSDD | Total (N=115)  Green care farm (N=34)  Traditional nursing home (N=29)  Regular small- scale living facility (N = 52) | M= 5.4 SD= 4.9  M= 5 SD= 4.4  M= 6.4 SD= 5.4  M= 5 SD= 4.9 |  |
| De Rooij et al. 2012 [18] | Caregiver relation (0–21) | Netherlands traditional (n=51)  Netherlands small-scale (n=51)  Belgium traditional (n=30)  Belgium small-scale (n=47) | 14.36  14.66  14.21  14.80 |  |
|  | Positive affect (0–18) | Netherlands traditional (n=51)  Netherlands small-scale (n=51)  Belgium traditional (n=30)  Belgium small-scale (n=47) | 10.85  14.12  11.87  12.56 | higher for residents in small-scale Dutch settings than in traditional Dutch settings  p=0.000 |
|  | Negative affect (0–9) | Netherlands traditional (n=51)  Netherlands small-scale (n=51)  Belgium traditional (n=30)  Belgium small-scale (n=47) | 5.97  5.54  4.59  6.00 | higher for residents in small-scale Belgium settings than in traditional Belgium settings  p=0.002 |
|  | Positive self-image (0–9) | Netherlands traditional (n=51)  Netherlands small-scale (n=51)  Belgium traditional (n=30)  Belgium small-scale (n=47) | 7.42  7.61  6.24  6.74 |  |
|  | Social relations (0–18) | Netherlands traditional (n=51)  Netherlands small-scale (n=51)  Belgium traditional (n=30)  Belgium small-scale (n=47) | 8.30  10.97  10.37  10.40 | higher for residents in small-scale Dutch settings than in traditional Dutch settings  p=0.000 |
|  | Social isolation (0–9) | Netherlands traditional (n=51)  Netherlands small-scale (n=51)  Belgium traditional (n=30)  Belgium small-scale (n=47) | 6.34  6.14  5.48  5.82 |  |
|  | Having something to do (0–6) | Netherlands traditional (n=51)  Netherlands small-scale (n=51)  Belgium traditional (n=30)  Belgium small-scale (n=47) | 0.91  2.43  1.58  2.11 | higher for residents in small-scale Dutch settings than in traditional Dutch settings  p=0.000 |
|  | Feeling at home (0–12) | Netherlands traditional (n=51)  Netherlands small-scale (n=51)  Belgium traditional (n=30)  Belgium small-scale (n=47) | 10.23  9.56  8.94  9.37 |  |
|  | Restless behaviour (0–9) | Netherlands traditional (n=51)  Netherlands small-scale (n=51)  Belgium traditional (n=30)  Belgium small-scale (n=47) | 4.63  5.11  4.30  3.81 |  |
|  | NPI-NH |  | No differences |  |
|  | Social engagement (0–8)  -> RISE | Netherlands traditional (n=51)  Netherlands small-scale (n=51)  Belgium traditional (n=30)  Belgium small-scale (n=47) | 2.43  3.69  2.52  2.71 | mean score was higher for the small-scale facilities than for traditional wards in Durch sample (p=0.004) |
|  | Visits (1–5) | Netherlands traditional (n=51)  Netherlands small-scale (n=51)  Belgium traditional (n=30)  Belgium small-scale (n=47) | 2.27  1.95  2.25  2.07 |  |
|  | Use of restraints (#) | Netherlands traditional (n=51)  Netherlands small-scale (n=51)  Belgium traditional (n=30)  Belgium small-scale (n=47) | 1.11  1.18  1.11  1.16 |  |
|  | Prescription of psychotropic medication (#) | Netherlands traditional (n=51)  Netherlands small-scale (n=51)  Belgium traditional (n=30)  Belgium small-scale (n=47) | 1.47  1.29  1.68  1.99 |  |
|  | Depression (0–38) | Netherlands traditional (n=51)  Netherlands small-scale (n=51)  Belgium traditional (n=30)  Belgium small-scale (n=47) | 8.45  8.37  10.27  8.50 | mean score on depressive symptoms (CSDD) was higher for traditional wards than for small-scale units (p=0.009) in Belgian sample |
|  | Behavioral problems (0–144) | Netherlands traditional (n=51)  Netherlands small-scale (n=51)  Belgium traditional (n=30)  Belgium small-scale (n=47) | 17.53  21.43  15.75  17.57 |  |
| Kok et al. 2018 [19] | QUALIDEM | Care relationship small-scale (t3)  Care relationship control group (t3)  Positive affect small-scale (T3)  Positive affect control group (T3)  Negative affect small-scale (T3)  Negative affect control group (T3)  Restless tense behavior small-scale (T3)  Restless tense behavior control group (T3)  Positive self image small-scale (T3)  Positive self image control group (T3)  Social relations small-scale (T3)  Social relations control group (T3)  Social isolation small-scale (T3)  Social isolation control group (T3)  Feeling at home small-scale (T3)  Feeling at home control group (T3)  Having something to do small-scale (T3)  Having something to do control group (T3) | M=13.3 SD=4.4  M=14.9 SD=4.1  M=11.8 SD=4.0  M=11.7 SD=4.9  M=6.0 SD=2.1  M=5.5 SD=2.4  M=4.3 SD=2.5  M=3.9 SD=2.7  M=6.7 SD=2.8  M=6.9 SD=2.7  M=8.6  M=8.5  M=5.9  M=5.9  M=9.5  M=10.6  M=1.2  M=1.1 | 0.158  0.158  0.955  0.955  0.813  0.813  0.541  0.541  0.377  0.377  0.415  0.415  0.273  0.273  0.235  0.235  0.951  0.951 |
|  | GDS-15 | Mood small-scale (T3)  Mood control (T3) | M=0.8  M=0.8 | 0.509  0.509 |
|  | GIP | Not social behavior small-scale (T3)  Not social control group (T3)  Apathy small-scale (T3)  Apathy control group (T3)  Insubordinate behavior small-scale (T3)  Insubordinate behavior control group (T3)  Suspicious behavior small-scale (T3)  Suspicious behavior control group (T3  Depressive behavior small-scale (T3)  Depressive behavior control group (T3)  Anxious behavior small-scale (T3)  Anxious behavior control group (T3) | M=21.1  M=20.6  M=15.3  M=15.4  M=9.8  M=10.0  M=8.7  M=9.5  M=9.3  M=9.1  M=7.9  M=9.7 | 0.650  0.650  0.902  0.902  0.617  0.617  0.222  0.222  0.635.  0.635  0.008* (significant)  0.008* (significant) |
| Smit et al. 2012 [20] | Task-related activities | Model 1  Constant  Group living characteristics  Number of residents at facility  Model 2  Constant  Group living characteristics  Number of residents at facility  Age  Sex (female)  NPI-Q neuropsychiatric symptoms  KATZ ADL dependency  R^2^ Model 1  R^2^ Model 2 | B=-2.716  SE=0.327  B=0.051***  SE=0.009  B=-0.002  SE=0.002  B=1.798  SE=0.932  B=0.047**  SE=0.009  B=-0.002  SE=0.002  B=-0.031**  SE=0.010  B=0.834*** SE=0.203  B=-0.007 SE=0.011  B=-0.471***  SE=0.048  B= 0*.*414  B=0.450 | p *<* 0.001  p *<* 0.01  p *<* 0.01  p *<* 0.001  p *<* 0.001 |
|  | Outdoor activities | Model 1  Constant  Group living characteristics  Number of residents at facility  Model 2  Constant  Group living characteristics  Number of residents at facility  Age  Sex (female)  NPI-Q neuropsychiatric symptoms  KATZ ADL dependency  R^2^ Model 1  R^2^ Model 2 | B=-2.381  SE=0.326  B=0.042***  SE=0.009  B=0.003  SE=0.002  B=1.526  SE=0.826  B=0.036***  SE=0.009  B=0.003  SE=0.002  B=-0.030***  SE=0.009  B=-0.360* SE=0.156  B=0.006 SE=0.010  B=-0.191***  SE=0.042  B=0.248  B=0.268 | p *<* 0.001  p *<* 0.001  p *<* 0.001  p < 0.05  p *<* 0.001 |
|  | Religion | Model 1  Constant  Group living characteristics  Number of residents at facility  Model 2  Constant  Group living characteristics  Number of residents at facility  Age  Sex (female)  NPI-Q neuropsychiatric symptoms  KATZ ADL dependency  R^2^ Model 1  R^2^ Model 2 | B=-1.569  SE=0.353  B=0.011  SE=0.009  B=-0.000  SE=0.003  B=-1.446  SE=0.896  B=0.007  SE=0.010  B=-0.000  SE=0.003  B=0.001  SE=0.009  B=0.561** SE=0.186  B=-0.021 SE=0.011  B=-0.051  SE=0.045  B=0.033  B=0.087 | p *<* 0.01 |
|  | Leisure | Model 1  Constant  Group living characteristics  Number of residents at facility  Model 2  Constant  Group living characteristics  Number of residents at facility  Age  Sex (female)  NPI-Q neuropsychiatric symptoms  KATZ ADL dependency  R^2^ Model 1  R^2^ Model 2 | B=-0.511  SE=0.275  B=0.031***  SE=0.007  B=-0.001  SE=0.002  B=2.940  SE=0.782  B=0.028***  SE=0.008  B=-0.001  SE=0.002  B=-0.020**  SE=0.008  B=0.071 SE=0.148  B=-0.044* SE=0.009  B=-0.222***  SE=0.043  B=0.175  B=0.225 | p *<* 0.001  p *<* 0.001  p *<* 0.01  p < 0.05  p < 0.001 |
|  | Physical exercise | Model 1  Constant  Group living characteristics  Number of residents at facility  Model 2  Constant  Group living characteristics  Number of residents at facility  Age  Sex (female)  NPI-Q neuropsychiatric symptoms  KATZ ADL dependency  R^2^ Model 1  R^2^ Model 2 | B=-2.402  SE=0.346  B=0.029**  SE=0.009  B=0.002  SE=0.002  B=2.014  SE=0.917  B=0.024**  SE=0.009  B=0.001  SE=0.002  B=-0.036***  SE=0.010  B=0.327 SE=0.189  B=-0.009 SE=0.011  B=-0.262***  SE=0.046  B=0.166  B=0.215 | p < 0.01  p < 0.01  p < 0.001  p < 0.001 |
|  | Creative activities | Model 1  Constant  Group living characteristics  Number of residents at facility  Model 2  Constant  Group living characteristics  Number of residents at facility  Age  Sex (female)  NPI-Q neuropsychiatric symptoms  KATZ ADL dependency  R^2^ Model 1  R^2^ Model 2 | B=-0.453  SE=0.253  B=0.012  SE=0.007  B=-0.002  SE=0.002  B=0.796  SE=0.743  B=0.011  SE=0.007  B=-0.002  SE=0.002  B=-0.012  SE=0.008  B=0.609 SE=0.146  B=-0.018* SE=0.009  B=-0.090*  SE=0.039  B=0.091  B=0.106 | p < 0.05  p < 0.05 |
|  | Intellectual activities | Model 1  Constant  Group living characteristics  Number of residents at facility  Model 2  Constant  Group living characteristics  Number of residents at facility  Age  Sex (female)  NPI-Q neuropsychiatric symptoms  KATZ ADL dependency  R^2^ Model 1  R^2^ Model 2 | B=-0.700  SE=0.265  B=0.013  SE=0.007  B=-0.010***  SE=0.002  B=1.029  SE=0.811  B=0.008  SE=0.008  B=-0.009***  SE=0.002  B=-0.002  SE=0.009  B=0.317* SE=0.160  B=-0.037*** SE=0.010  B=-0.239***  SE=0.041  B=0.346  B=0.084 | p < 0.001  p < 0.001  p < 0.05  p < 0.001  p < 0.001 |
|  | Activities with senses | Model 1  Constant  Group living characteristics  Number of residents at facility  Model 2  Constant  Group living characteristics  Number of residents at facility  Age  Sex (female)  NPI-Q neuropsychiatric symptoms  KATZ ADL dependency  R^2^ Model 1  R^2^ Model 2 | B=-1.104  SE=0.268  B=0.004  SE=0.007  B=-0.001  SE=0.002  B=-2.327  SE=0.829  B=0.006  SE=0.007  B=-0.002  SE=0.002  B=-0.005  SE=0.009  B=1.147*** SE=0.196  B=-0.007 SE=0.010  B=0.144**  SE=0.046  B=0.027  B=0.142 | p < 0.001  p < 0.01 |
|  | Interaction with others | Model 1  Constant  Group living characteristics  Number of residents at facility  Model 2  Constant  Group living characteristics  Number of residents at facility  Age  Sex (female)  NPI-Q neuropsychiatric symptoms  KATZ ADL dependency  R^2^ Model 1  R^2^ Model 2 | B=-0.377  SE=0.263  B=0.031***  SE=0.007  B=-0.002  SE=0.002  B=0.839  SE=0.777  B=0.028***  SE=0.008  B=-0.002  SE=0.002  B=-0.007  SE=0.008  B=0.054 SE=0.149  B=-0.013 SE=0.009  B=-0.291***  SE=0.044  B=0.229  B=0.000 | p < 0.001  p < 0.001  p < 0.001 |
| Te Boekhorst et al. 2009 [21] | MMSE | Upon admission:  Nursing homes (n=97)  Group living homes (n=67)  6 month after admission:  Nursing homes (n=97)  Group living homes (n=67) | M (95%-CI)  10.3 (8.3-12.3)  15.4 (13.5-17.3)  8.9 (6.2-11.6)  13.0 (10.4-15.6) | No significant differences between groups. |
|  | IDDD | Upon admission:  Nursing homes (n=97)  Group living homes (n=67)  6 month after admission:  Nursing homes (n=97)  Group living homes (n=67) | M (95%-CI)  33.0 (30.5-35.6)  25.9 (22.9-28.8)  34.6 (31.9-37.2)  28.3 (26.3-30.3) | indicates decline for both groups. Significance of adjusted regression coefficient shows that deterioration was less pronounced in GL residents |
|  | RMBPC | Upon admission:  **MBPC Memory**  Nursing homes (n=97)  Group living homes (n=67)  **MBPC Depression**  Nursing homes (n=97)  Group living homes (n=67)  **RMBPC Behavior**  Nursing homes (n=97)  Group living homes (n=67)  6 month after admission:  **RMBPC Memory**  Nursing homes (n=97)  Group living homes (n=67)  **RMBPC Depression**  Nursing homes (n=97)  Group living homes (n=67)  **RMBPC Behavior**  Nursing homes (n=97)  Group living homes (n=67) | M (95%-CI)  21.6 (21.0-22.3)  20.8 (19.9-21.7)  13.1 (12.3-13.8)  14.9 (12.8-17.0)  6.7 (6.0-7.4)  6.1 (4.9-7.3)  17.2 (14.8-19.7)  15.8 (14.3-17.3)  8.0 (7.4-8.6)  8.9 (7.4-10.5)  5.4 (4.7-6.0)  4.5 (3.5-5.4) | No significant differences between groups. |
|  | NPI-Q | Upon admission:  Nursing homes (n=97)  Group living homes (n=67)  6 month after admission:  Nursing homes (n=97)  Group living homes (n=67) | M (95%-CI)  11.7 (10.9-12.8)  12.1 (10.5-13.8)  8.8 (7.5-10.1)  7.5 (6.2-8.7) | No significant differences between the groups. |
|  | RISE from RAI | Upon admission:  Nursing homes (n=97)  Group living homes (n=67)  6 month after admission:  Nursing homes (n=97)  Group living homes (n=67) | M (95%-CI)  2.9 (2.5-3.2)  3.2 (2.7-3.7)  3.2 (2.6-3.7)  4.5 (4.0-5.0) | Univariate regression analysis: residents of group living homes significantly more socially engaged at t2 |
|  | DQoL | 6 month after admission:  **Sense of aesthetics**  Nursing homes (n=97)  Group living homes (n=67)  **Self-esteem**  Nursing homes (n=97)  Group living homes (n=67)  **Positive affect**  Nursing homes (n=97)  Group living homes (n=67)  **Negative affect**  Nursing homes (n=97)  Group living homes (n=67)  **Feelings of belonging**  Nursing homes (n=97)  Group living homes (n=67)  **Overall quality of life**  Nursing homes (n=97)  Group living homes (n=67) | M (95%-CI)  7.1 (5.2-8.9)  10.8 (9.5-12.2)  6.6 (5.0-8.1)  7.8 (6.8-8.8)  12.1 (11.2-13.0)  13.7 (12.3-15.1)  16.9 (14.3-19.6)  18.6 (16.6-20.5)  5.5 (4.7-6.3)   - 1. (5.8-7.4)   2.0 (1.8-2-2)  2.3 (2.0-2.6) | Significant difference: “sense of aesthetics” (p<0.05) |
|  | QUALIDEM | 6 month after admission:  **Care relationship**  Nursing homes (n=97)  Group living homes (n=67)  **Positive affect**  Nursing homes (n=97)  Group living homes (n=67)  **Negative affect**  Nursing homes (n=97)  Group living homes (n=67)  **Restless tense Behavior**  Nursing homes (n=97)  Group living homes (n=67)  **Social relations**  Nursing homes (n=97)  Group living homes (n=67)  **Having something to do**  Nursing homes (n=97)  Group living homes (n=67) | M (95%-CI)  6.1 (5.6-6.6)  5.3 (4.2-6.4)  4.7 (4.0-5.4)  4.0 (3.1-4.8)  3.4 (2.7-4.2)  3.5 (3.0-4.0)  3.5 (2.1-4.7)  3.4 (2.4-4.2)  7.3 (5.7-8.9)  4.8 (3.4-6.1)  1.9 (1.3-2.7)  4.3 (3.8-4.8) | Significant difference:  “Having something to do” p<0.001 |
| Verbeek et al. 2010 [22] | QUALIDEM, Residents  total score | Small-scale - Care relationships  T0 (baseline)  T1 (Follow-up, 6 mo)  T2 (Followup, 12 mo)  Regular ward - Care relationships  T0  T1  T2 | 15.0 (SD=4.5)  14.6 (4.4)  14.5 (4.4)  15.5 (4.6)  15.8 (4.8)  15.8 (4.6) |  |
|  |  | Small-scale - Positive affect  T0  T1  T2  Regular ward - Positive affect  T0  T1  T2 | 14.5 (3.7)  13.8 (3.9)  13.7 (3.7)  14.0 (4.0)  13.8 (3.7)  13.7 (4.1) |  |
|  |  | Small-scale - Negative affect  T0  T1  T2  Regular ward - Negative affect  T0  T1  T2 | 6.6 (1.9)  6.6 (2.3)  6.6 (2.4)  5.8 (2.2)  5.7 (2.4)  5.7 (2.3) | Group effects found on subscales scored  by nursing staff: Residents in EG had lower QoL regarding negative affect than C residents  (0.7, 0.2–1.2; P = 0.01) |
|  |  | Small-scale - Restless behavior  T0  T1  T2  Regular ward - Restless behavior  T0  T1  T2 | 5.4 (2.8)  5.3 (2.8)  4.8 (2.8)  5.7 (2.7)  5.5 (3.0)  5.8 (2.8) |  |
|  |  | Small-scale - Social isolation  T0  T1  T2  Regular ward - Social isolation  T0  T1  T2 | 6.6 (2.0)  6.4 (2.3)  6.1 (1.9)  6.8 (2.3)  6.8 (2.3)  6.9 (2.2) |  |
|  |  | Small-scale - Positive self-image  T0  T1  T2  Regular ward - Positive self-image  T0  T1  T2 | 7.1 (2.1)  7.2 (2.3)  7.1 (2.2)  7.4 (2.1)  7.6 (2.1)  7.7 (2.0) |  |
|  |  | Small-scale – Having something to do  T0  T1  T2  Regular ward - Having something to do  T0  T1  T2 | 2.7 (2.1)  2.3 (2.0)  2.2 (2.0)  1.9 (2.0)  1.6 (1.7)  1.4 (1.6) | Group effects found on subscales scored  by nursing staff: Residents in EG had higher QoL with respect  to having something to do (adjusted mean difference  0.9, 95% confidence interval [CI] 0.5–1.2; P < .001) |
|  |  | Small-cale – Feeling at home  T0  T1  T2  Regular ward - Feeling at home  T0  T1  T2 | 9.7 (2.7)  9.8 (2.5)  9.5 (2.9)  9.8 (2.6)  10.1 (2.4)  10.4 (2.0) | Group effects found on subscales scored by family caregivers: Residents in EG had higher QoL on feeling at home ((1.0, 0.1–2.0; P = .023). Group effects scored by family caregivers: 1.0, 0.1–2.0; P = .023. |
|  |  | Small-cale – Social relations  T0  T1  T2  Regular ward – Social relations  T0  T1  T2 | 12.4 (3.8)  10.7 (3.9)  11.0 (3.9)  11.3 (4.1)  11.6 (3.8)  10.3 (3.7) | Group effects were found on subscales scored by family caregivers: Residents in EG had higher QoL on social relations (1.1, 0.2–2.0;  P = .02) |
|  | Mean total QUALIDEM score | Small-cale  T0  T1  T2  Regular ward  T0  T1  T2 | 18.8 (3.5)  18.3 (3.7)  17.5 (3.8)  18.8 (3.8)  18.6 (3.9)  18.4 (3.6) |  |
|  | Total score NPI-NH | Small-cale  T0  T1  T2  Regular ward  T0  T1  T2 | 16.2 (14.0)  13.5 (12.0)  16.6 (14.9)  15.7 (13.7)  14.3 (12.7)  14.8 (12.1) |  |
|  | Total score CMAI | Small-cale  T0  T1  T2  Regular ward  T0  T1  T2 | 40.3 (14.2)  37.2 (11.5)  39.5 (13.5)  40.6 (14.0)  38.5 (11.7)  35.3 (8.0) |  |
| Verbeek et al. 2014 [23] | CMAI | Physically non-aggressive behavior  Small-scale  Baseline  6 months  12 months  Regular ward  Baseline  6 months  12 months  Physically aggressive behavior  Small-scale  Baseline  6 months  12 months  Regular ward  Baseline  6 months  12 months  Verbally agitated behavior  Small-scale  Baseline  6 months  12 months  Regular ward  Baseline  6 months  12 months | 10.75 (5.72)  10.39 (5.62)  11.09 (6.34)  10.50 (5.85)  9.45 (4.27)  8.42 (3.13)  10.47 (4.93)  10.14 (3.72)  10.38 (4.67)  11.53 (5.54)  10.28 (4.71)  9.93 (3.59)  7.90 (5.23)  6.62 (4.47)  6.82 (4.93)  7.28 (3.92)  6.02 (2.92)  5.82 (3.43) |  |
|  | NPI-NH | Delusions  Small-scale  Baseline  6 months  12 months  Regular ward  Baseline  6 months  12 months  Hallucinations  Small-scale  Baseline  6 months  12 months  Regular ward  Baseline  6 months  12 months  Agitation/aggression  Small-scale  Baseline  6 months  12 months  Regular ward  Baseline  6 months  12 months  Depression  Small-scale  Baseline  6 months  12 months  Regular ward  Baseline  6 months  12 months  Anxiety  Small-scale  Baseline  6 months  12 months  Regular ward  Baseline  6 months  12 months  Euphoria  Small-scale  Baseline  6 months  12 months  Regular ward  Baseline  6 months  12 months  Apathy  Small-scale  Baseline  6 months  12 months  Regular ward  Baseline  6 months  12 months  Disinhibition  Small-scale  Baseline  6 months  12 months  Regular ward  Baseline  6 months  12 months  Irritability  Small-scale  Baseline  6 months  12 months  Regular ward  Baseline  6 months  12 months  Abberant motor behavior  Small-scale  Baseline  6 months  12 months  Regular ward  Baseline  6 months  12 months  Night-time behavior  Small-scale  Baseline  6 months  12 months  Regular ward  Baseline  6 months  12 months  Eating change  Small-scale  Baseline  6 months  12 months  Regular ward  Baseline  6 months  12 months | 1.18 (2.95)  0.86 (2.37)  1.34 (3.14)  1.07 (2.61)  1.14 (2.62)  1.21 (2.48)  0.86 (2.35)  0.57 (1.54)  0.88 (2.34)  0.79 (2.25)  0.57 (1.90)  0.46 (1.63)  1.90 (3.05)  1.14 (2.21)  1.45 (2.87)  2.10 (3.20)  1.77 (3.02)  2.00 (3.13)  1.98 (2.99)  1.99 (3.22)  1.88 (3.35)  1.67 (3.03)  1.51 (2.81)  1.55 (2.72)  1.03 (2.49)  1.00 (2.66)  0.97 (2.62)  1.16 (2.62)  0.89 (2.22)  1.37 (2.72)  0.30 (1.22)  0.14 (1.76)  0.37 (1.59)  0.38 (1.43)  0.26 (1.05)  0.11 (.58)  2.06 (3.27)  1.88 (3.10)  2.75 (3.65)  2.29 (3.68)  2.43 (3.40)  2.46 (3.68)  1.27 (2.56)  0.65 (1.69)  1.15 (2.63)  1.09 (2.49)  0.79 (2.25)  0.63 (2.01)  2.12 (3.07)  2.28 (3.10)  2.37 (3.28)  2.00 (2.91)  1.65 (2.77)  1.88 (3.27)  1.55 (3.18)  1.48 (2.81)  1.60 (2.94)  0.81 (2.31)  1.17 (2.63)  0.99 (2.72)  1.02 (2.46)  0.64 (1.71)  0.97 (2.20)  1.10 (2.48)  0.66 (1.82)  0.97 (2.17)  0.94 (2.55)  1.06 (2.87)  1.42 (3.42)  1.21 (3.17)  1.40 (3.29)  1.10 (2.77) |  |
|  | CSDD | Small-scale  Baseline  6 months  12 months  Regular ward  Baseline  6 months  12 months | 5.49 (4.75)  4.24 (4.24)  3.46 (4.71)  5.25 (4.53)  3.41 (2.85)  3.28 (4.06) |  |

AARS: Apparent Affect Rating Scale; ADL: activities of daily living; BCRS: Brief Cognitive Rating Scale; CG: comparison group; CMAI: Cohen Mansfield Agitation Inventory; CSDD: Cornell Scale for Depression; CPS: Cognitive performance scale; DAD: Disability Assessment for Dementia; DD: Depression in Dementia scale; DQoL: Dementia Quality of Life instrument; EG: experimental group; FAM: Functional Assessment Measure; FIM: Functional Independence Measure; FAST: Functional Assessment Staging; GBS: Gottfries-Bråne-Steen scale; GDS: Global Deterioration Scale; GDS-15: Geriatric Depression Scale; GIP: Gedragsobservatieschaal voor Intramurale Psychogeriatrie; GL: group living; GLM: generalized linear model; IADL: instrumental activities of daily living; IDDD: Interview for the Deterioration of Daily Living activities in Dementia; IRR: Incidence rate ratio; M: mean; MDS: Minimum data set; MMSE: Mini Mental State Examination; Mo: months; MOSES: Multidimensional Observation Scale for Elderly Subjects; MSS: Mood scale sore; NH: nursing home; NPI-NH: Neuropsychiatric Inventory – Nursing Home version; NPI-Q: Neuropsychiatric Inventory-Questionnaire; n.s.: not significant; OR: odds ratio; QoL: quality of life; QoL-AD: Quality of Life in Alzheimer's Disease; RCC: Residential care center; RMBPC: Revised Memory and Behavior Problems Checklist; RISE: Revised Index for Social Engagement; SE: standard error; SD: standard deviation; SCU: special care unit; SHA: shared-housing arrangement; QoL: quality of life

**References**

1. Kane RA, Lum TY, Cutler LJ, Degenholtz HB, Yu TC. Resident outcomes in small-house nursing homes: a longitudinal evaluation of the initial green house program. J Am Geriatr Soc. 2007 Jun;55(6):832-9.
2. Kane RA, Kling KC, Bershadsky B, Kane RL, Giles K, Degenholtz HB, Liu J, Cutler LJ. Quality of life measures for nursing home residents. J Gerontol A Biol Sci Med Sci. 2003 Mar;58(3):240-8.
3. Yoon, JY, Brown RL, Bowers BJ, Sharkey SS, Horn SD. Longitudinal Psychological Outcomes of the Small-scale Nursing Home Model: a Latent Growth Curve Zero-inflated Poisson Model. Int Psychogeriatr. 2015 Jun; 27(6): 1009–1016.
4. Yoon JY, Brown RL, Bowers BJ, Sharkey SS, Horn SD. The effects of the green house nursing home model on ADL function trajectory: a retrospective longitudinal study. Int J Nurs Stud. 2016 Jan;53:238-47.
5. Molony S, Evans LK, Jeon S, Rabig J, Straka LA. Trajectories of at-homeness and health in usual care and small house nursing homes. Gerontologist. 2011 Aug;51(4):504-15.
6. Annerstedt L. An attempt to determine the impact of group living care in comparison to traditional long-term care on demented elderly patients. Aging (Milano). 1994 Oct;6(5):372-80.
7. Kihlgren M, Bråne G, Karlsson I, Kuremyr D, Leissner P, Norberg A. Long-Term Influences on Demented Patients in Different Caring Milieus, a Collective Living Unit and a Nursing Home: A Descriptive Study. Dementia 1992;3:342-349.
8. Nyth AL, Bråne G. Principal Component Analysis of the GBS Scale. Dementia 1992;3:193–199
9. Wimo A, Adolfsson R, Sandman P. Care for demented patients in different living conditions: Effects on cognitive function, ADL capacity and behavior. Scandinavian Journal of Primary Health Care. 1995;13:3,205-10.
10. Ritchie K, Ledésert B. The measurement of incapacity in the severely demented elderly: The validation of a behavioural assessment scale. Int J Geriatric Psychiat. 1992; 6, 217-266.
11. Suzuki M, Kanamori ;. Yasuda M, Oshiro H. One-year follow-up study of elderly group-home residents with dementia. Am J Alzheimers Dis Other Demen. Aug-Sep 2008;23(4):334-43.
12. Auer S, Kienberger U, Pascher P, Geck M, Hoffmann B, Viereckl C, Span E. Wohngemeinschaft versus traditionelles Pflegeheim für Personen mit Demenz-Eine vergleichende Beobachtungsstudie. Pflegewissenschaft. 2017;19(3):156-165.
13. Reimer MA, Slaughter S, Donaldson C, Currie G, Eliasziw M. Special Care Facility Compared with Traditional Environments for Dementia Care: A Longitudinal Study of Quality of Life. J Am Geriatr Soc. 2004;52:1085–1092.
14. Wolf-Ostermann K, Worch A, Fischer T, Wulff I, Gräske J. Health outcomes and quality of life of residents of shared-housing arrangements compared to residents of special care units - results of the Berlin DeWeGE-study. J Clin Nurs. 2012a;21(21-22):3047-60.
15. Dettbarn-Reggentin J. Studie zum Einfluss von Wohngruppenmilieus auf demenziell Erkrankte in stationären Einrichtungen. Zeitschrift für Gerontologie und Geriatrie 2005;38,95–100.
16. Warren S, Janzen W, Andiel-Hett C, Liu L, McKim HR, Schalm C. Innovative Dementia Care: Functional Status over Time of Persons with Alzheimer Disease in a Residential Care Centre Compared to Special Care Units. Dement Geriatr Cogn Disord, 2001;12:340–347.
17. De Boer B, Hamers JPH, Zwakhalen SMG, Tan FES, Beerens HC, Verbeek H. Green Care Farms as Innovative Nursing Homes, Promoting Activities and Social Interaction for People With Dementia. J Am Med Dir Assoc. 2017b;18(1):40-46.
18. De Rooij AHPM, Luijkx KG, Schaafsma J, Declerq AG, Emmerink PMJ, Schols JMGA. Quality of life of residents with dementia in traditional versus small-scale long-term care settings: a quasi-experimental study. Int J Nurs Stud. 2012 Aug;49(8):931-40.
19. Kok JS, Nielen MMA, Scherder EJA. Quality of life in small-scaled homelike nursing homes: an 8-month controlled trial. Health Qual Life Outcomes. 2018 Feb 27;16(1):38.
20. Smit D, de Lange J, Willemse B, Pot AM. The relationship between small-scale care and activity involvement of residents with dementia. Int Psychogeriatr. 2012 May;24(5):722-32.
21. Te Boekhorst S, Depla MFIA, de Lange J, Pot AMP, Eefsting JA. The effects of group living homes on older people with dementia: a comparison with traditional nursing home care. Int J Geriatr Psychiatry. 2009 Sep;24(9):970-8.
22. Verbeek H, Zwakhalen SMG, van Rossum E, Ambergen T, Kempen GIJM, Hamers JPH. Dementia care redesigned: Effects of small-scale living facilities on residents, their family caregivers, and staff. J Am Med Dir Assoc. 2010 Nov;11(9):662-70.
23. Verbeek H, Zwakhalen SM, Van Rossum E, Ambergen T, Kempen GIJM, Hamers JPH. Effects of small-scale, home-like facilities in dementia care on residents’ behavior, and use of physical restraints and psychotropic drugs: a quasi-experimental study. Int Psychogeriatr. 2014 Apr;26(4):657-68.
